# Path analysis for controlling climate change in global aviation

**DOI:** 10.1016/j.isci.2024.110126

**Published:** 2024-05-28

**Authors:** Qiang Cui, Yi-lin Lei, Zi-ke Jia, Yu Wang, Ye Li

**Affiliations:** 1 School of Economics and Management, Southeast University, Nanjing, China; 2 School of Economics and Management, Civil Aviation Flight University of China, Guanghan, China; 3 School of Business Administration, Nanjing University of Finance and Economics, Nanjing, China

**Keywords:** Environmental science, Global change, Energy policy, Global carbon cycle, Aviation

## Abstract

The aviation industry’s emissions have had a significant impact on global climate change. This study focuses on carbon emission trading schemes, sustainable aviation fuels (SAFs), and hydrogen energy, as vital means for the aviation industry to reduce emissions. To evaluate the climate effects of global routes under four scenarios (24 sub-scenarios) until 2100, this study proposes the Aviation-FAIR (Aviation-Finite Amplitude Impulse Response) method. The findings reveal that while CO_2_ emissions and concentrations are significant, other emissions, such as N_2_O and CH_4_, have a greater effective radiative forcing (ERF) and contribute significantly to climate change. Moreover, SAFs are more effective in mitigating airline pollutant emissions than relying solely on carbon trading schemes. The effectiveness of hydrogen fuel cells may be hindered by technical limitations compared to hydrogen turbine engines. The findings of this study provide reference for the global aviation industry to adopt emission reduction measures.

## Highlights

• Collects the CO_2_, CO, HC, NOx, PM2.5, and SO_2_ emissions from global routes
• The FAIR method is systematically designed for aviation emissions
• Compare the carbon emission trading schemes and sustainable aviation fuel
• Combine hydrogen turbines and hydrogen fuel cells with biomass fuel

## Graphical abstract

**Figure:**
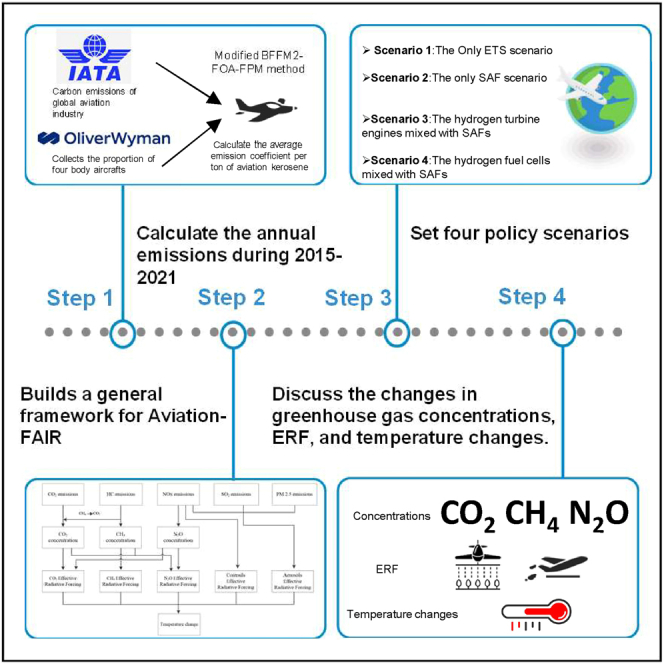

## Introduction

Since the onset of the 21st century, the aviation industry has experienced rapid growth, emerging as a pivotal force driving economic development.^1^^,^^2^ However, this expansion has brought about an increased reliance on fossil energy, leading to a continual upswing in annual aviation carbon emissions. In 2019, the global civil aviation sector contributed 918 million tons of carbon dioxide to the atmosphere, marking a 29% surge from 2013 and constituting roughly 2% of worldwide emissions.^3^ The combustion of fossil kerosene during flight operations emerges as a predominant source, emitting an estimated 2.5 kg of carbon dioxide per liter.^4^ Owing to the absence of mitigating strategies, aviation carbon emissions are projected to represent 10% of global greenhouse gas emissions by 2050.^5^ Due to the excessive emission of gases, including carbon dioxide, from the aviation industry, it will further lead to the formation of a “greenhouse effect enhancement layer,” exacerbating the Earth’s heat retention and resulting in global warming.^6^ Therefore, these data underscore the significant role aviation plays in climate change, emphasizing the enduring upward trajectory of aviation emissions and their escalating impact on global warming. Consequently, advocating for reductions in aviation emissions becomes imperative for fostering global climate equity.

To curb the escalation of aviation carbon emissions, global initiatives are being implemented, encompassing measures like carbon trading, the adoption of sustainable aviation fuel (SAF), and the integration of hydrogen energy to address these environmental challenges.^7^^,^^8^^,^^9^^,^^10^ In terms of carbon trading, launched in 2005, the EU (European Union) emissions trading system (EU ETS) is dedicated to fulfilling the emission reduction objectives outlined in the Kyoto Protocol. Mayor’s research suggests that an aviation carbon tax may effectively decrease domestic carbon emissions in the presence of substitution effects from domestic and international tourism. In-depth analysis of airfare reveals that aviation carbon taxes result in heightened ticket prices, subsequently curbing air travel demand and ultimately realizing a carbon emissions reduction ranging from 9% to 32%.^11^

However, the EU ETS offers economic compensation for aviation emissions’ environmental impact but falls short of achieving genuine carbon emission reductions. Hence, the pursuit of aviation clean energy is pivotal. Biomass, electric, hydrogen, and hybrid power emerge as primary clean energy options. SAFs, an “alternative” to fossil fuels, align with ICAO (International Civil Aviation Organization) sustainability standards,^12^ drawing scholarly attention. Recent studies focus on exploring production challenges, strategies, emission reduction potential, utilization, costs, and benefits of SAF. While biofuels exhibit higher technological maturity than other alternatives, their sustainable large-scale production presents significant challenges.^13^ Besides, achieving carbon neutrality in the future demands a synergistic approach that combines upcoming aircraft technologies with alternative fuels.^14^ On the other hand, hydrogen, with three times the energy density and 11 times the specific density of traditional fuels,^15^^,^^16^ emerges as a cleaner and more sustainable aviation alternative devoid of carbon emissions.^17^^,^^18^^,^^19^^,^^20^ The increasing demand for hydrogen in aviation has spurred research, highlighting its global availability, safety, low pollution, and lightweightness.^21^ Recognized as a viable aircraft energy carrier, scholars predict hydrogen consumption for aviation,^22^^,^^23^^,^^24^ particularly focusing on hydrogen fuel engines and fuel cell systems in hydrogen aviation power technology.^25^^,^^26^ This has prompted an evaluation of the feasibility of promoting hydrogen fuel usage in aircraft.^27^^,^^28^

In the realm of climate change research models, simple models offer a valuable tool for simulating the responses of radiative forcing and temperature to emissions and atmospheric concentrations. They can be fine-tuned to emulate the behavior of a single climate and Earth system model, as observed in various studies.^29^^,^^30^^,^^31^^,^^32^^,^^33^ However, limitations emerge in these models as they fail to fully capture the temporal evolution dependence of carbon sinks under diverse background conditions.^34^^,^^35^ To address this gap, the Finite Amplitude Impulse Response (FAIR) model was introduced. This model tracks the time integral fraction of carbon in the air, determines the efficiency of carbon sinks, and subsequently computes changes in carbon dioxide concentration, radiative forcing, and atmospheric temperature. In comparison to alternative climate change research models, FAIR v1.0 stands out for its well-calibrated representation of the temperature and carbon cycle responses within the Earth system model. The subsequent iteration, FAIR v1.3, expands its capabilities to calculate the concentration of non-CO_2_ greenhouse gases.^36^^,^^37^ This evolution enhances the model’s comprehensiveness and applicability in the study of climate change dynamics.

**Figure 1:**
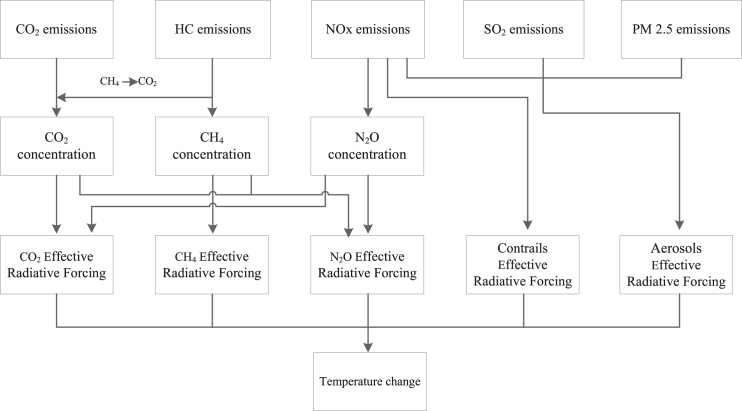
Framework of Aviation-FAIR

In summary, the prevailing research on the influence of aviation pollutants on climate change exhibits notable deficiencies. Firstly, there is a scarcity of comprehensive studies addressing the two primary avenues for aviation emission reduction—namely, SAFs and carbon trading schemes. Existing research tends to concentrate solely on one of these approaches, lacking a comparative analysis. Secondly, there is an absence of systematic investigation into the utilization of hydrogen energy, specifically liquid hydrogen, within the aviation sector. This study, considering the application scenarios of biomass fuels, examines the climate change impact resulting from the integration of hydrogen turbine engines and hydrogen fuel cells with biomass fuels. Lastly, the FAIR method, although not originally tailored for aviation emissions, becomes a focal point in this study, which introduces an Aviation-FAIR framework to address this specific issue, as shown in Figure 1.

## Results

This study sets four scenarios: the baseline scenario (scenario 0), the scenario of only an ETS (scenario 1), the scenario of only SAFs (scenario 2), and the scenario of hydrogen energy mixed with SAFs (the details can be found in the supplementary information). The last one contains two sub-scenarios: a hydrogen turbine engine (HT) mixed with SAFs (scenario 3) and a hydrogen fuel cell (HFC) mixed with SAFs (scenario 4). Each scenario has several sub-scenarios (see details in the supplementary information). This study has used the Aviation-FAIR method (see details in the Method section) to compare the concentration changes, the trend of effective radiative forcing (ERF), and temperature changes until 2100 under these scenarios. This study has discussed the differences between these scenarios in terms of concentration changes, ERF, and temperature. The detailed scenarios can be found in the supplementary information.

### Concentration changes in the five scenarios of global aviation

**Figure 2:**
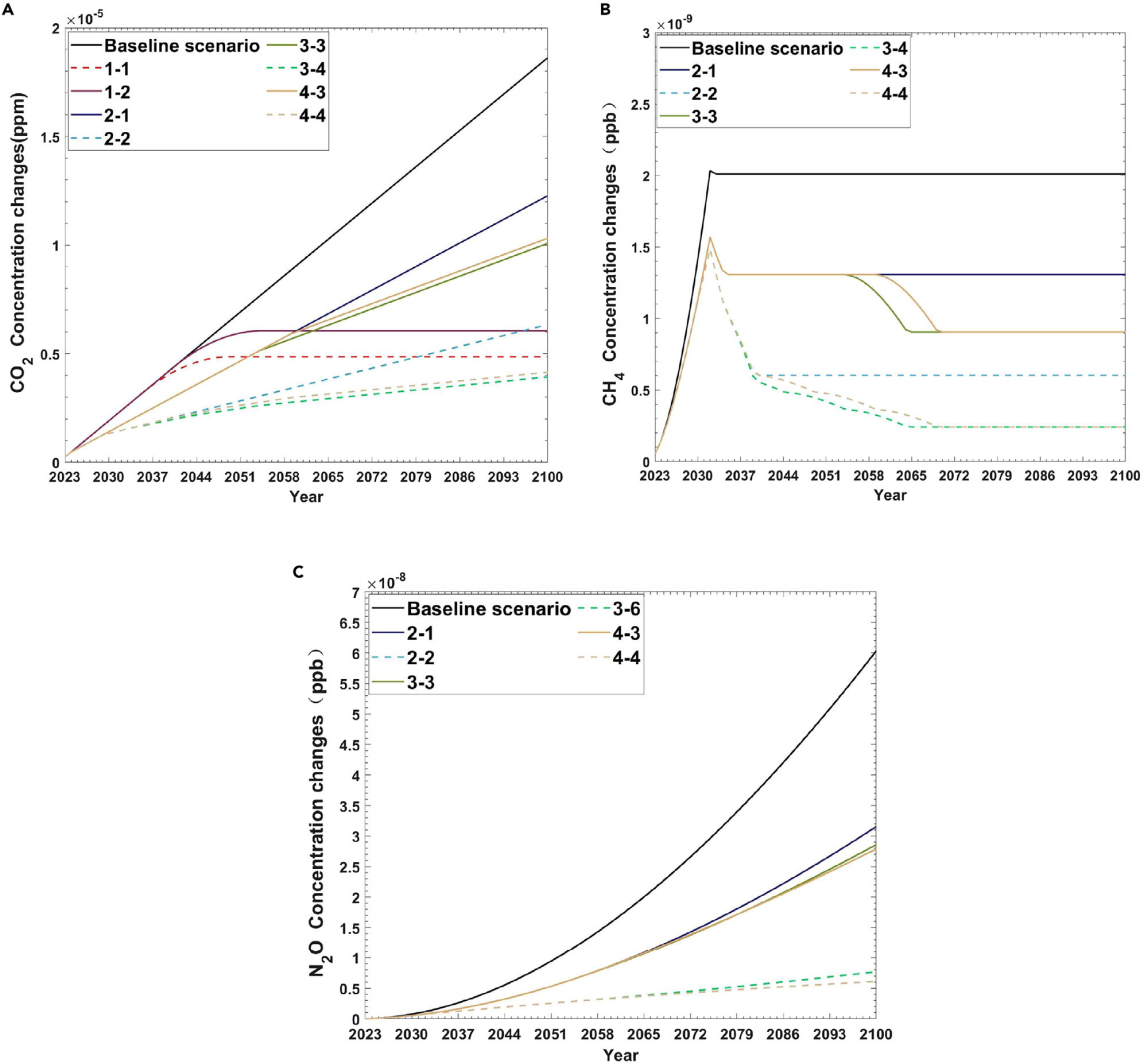
Concentration changes of CO_2_, N_2_O and CH_4_ under five scenarios from 2023 to 2100 (A) Concentration of CO_2_. (B) Concentration of CH_4_. (C) Concentration of N_2_O.

This study compares the changes in CO_2_, CH_4_, and N_2_O concentrations from 2023 to 2100 under the four scenarios. As mentioned in the supplementary information, unrestricted climate impact refers to the impact of global aviation pollutant emissions on climate without carbon emission reduction measures such as carbon trading scheme regulation and the use of SAF. Figure 2 shows the concentration changes of CO_2_, CH_4_, and N_2_O under five scenarios from 2023 to 2100. From scenario 1 to scenario 4, the solid line represents the sub-scenario with the most minor change compared to the baseline scenario. In contrast, the dashed line represents the sub-scenario with the most considerable change compared to the baseline scenario.

By comparing the concentration changes of three pollutants, this study can find that the impact of CO_2_ on the greenhouse effect remains the greatest. If not controlled, in the baseline scenario, by 2100, the concentration will increase by 1.86E-05 ppm, ranking second in N_2_O concentration.

Figure 2A shows the concentration changes of CO_2_ from 2023 to 2100. The concentrations of CO_2_ in the five scenarios show an upward trend. Among them, scenario 1, scenario 2, scenario 3, and scenario 4, the sub-scenario with the slightest change in carbon dioxide concentration (1-2, 2-1, 3, and 4-3) also shows a significant decrease compared to the baseline scenario, indicating that all four scenarios have reasonable control over CO_2_ concentration. The most significant performance is in the nine scenarios of the hydrogen turbine engines mixed with SAFs (scenario 3). In the case of the baseline scenario, compared with 2022, the growth of CO_2_ concentration will be linearly and incrementally by 1.86E-05 ppm by 2100. However, in scenario 3-4, by 2100, the minimum CO_2_ concentration is only 3.93E-06 ppm, approximately 21.12% of the baseline scenario. Under nine scenarios of the hydrogen fuel cells mixed with SAFs (scenario 4), by 2100, its CO_2_ concentration range is 4.15E-06 ppm −1.03E-05 ppm, second only to scenario 3. Next are the only ETS scenarios (scenario 1) and the only SAF scenario (scenario 2), where CO_2_ control is less effective than improving the aircraft engine. In the only ETS scenarios (scenario 1) and the only SAF scenario (scenario 2), by 2100, the CO_2_ concentration ranges are 6.05E-06ppm–4.86E-06ppm and 6.34E-06ppm–1.23E-05ppm, respectively. It is worth noting that both scenarios under the only ETS scenario have better CO_2_ control than the only SAF scenario. If the peak of evaporation carbon can be achieved in 2035, the growth of CO_2_ concentration under the 2035–2050 scenario (scenario 1-1) will be 4.86E-06 ppm by 2100, which is lower than the three sub-scenarios with only SAF scenarios (scenario 2).

Figures 2B and 2C show the concentration changes of CH_4_ and N_2_O from 2023 to 2100, respectively. Among them, compared with the baseline scenario, the control of CH_4_ and N_2_O concentrations under the only ETS scenarios is insignificant, which is not shown in the figure. As shown in Figure 2B, the two most significant scenarios for CH_4_ control are still scenario 3 and scenario 4. By 2100, the minimum CH_4_ concentration is only 2.41E-10ppb (scenario 3-4 (2025&SAF_50%_ + 2030&SAF_100%_ + 2035&HT_20%_&SAF_80%_ + 2045&HT_40%_&SAF_60%_ + 2055&HT_60%_&SAF_40%_), about 12% of the baseline scenario. Next is the only SAF scenarios. By 2100, the CH_4_ concentration reaches 6.03E-10ppb–1.31E-09ppb, indicating that the control effect of CH_4_ under the three sub-scenarios is not as good as scenario 3 and scenario 4.

And the concentrations of N_2_O in the five scenarios also show an upward trend. As shown in Figure 2C, compared with the baseline scenario, only SAF scenario (scenario 2), the hydrogen turbine engines mixed with SAFs (scenario 3), and the hydrogen fuel cells mixed with SAFs (scenario 4) have significant control over N_2_O, but the difference is not significant. By 2100, the minimum N_2_O concentrations are 6.12E-09ppb, 7.71E-09ppb, and 6.12E-09ppb, respectively, all below the baseline scenario (6.04E-08 ppb). However, it is worth noting that different from CO_2_ concentration and CH_4_ concentration, the control effect on N_2_O concentration in scenario 3 is not as good as in Scenario 4.

### ERF changes in the five scenarios of global aviation

**Figure 3:**
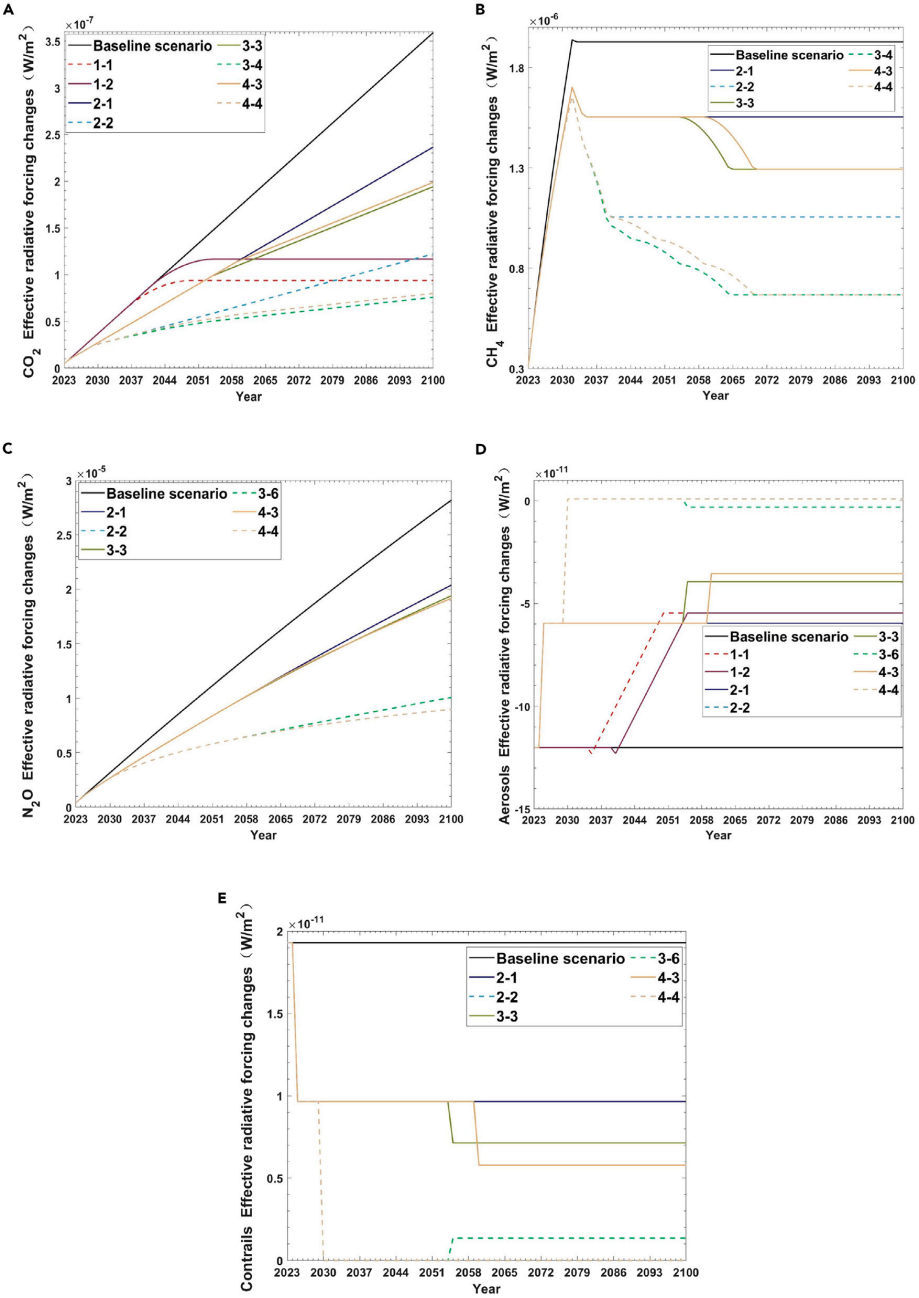
Effective Radiative Forcing (ERF) changes of CO_2_, CH_4_, N_2_O, contrails and aerosol under five scenarios from 2023 to 2100 (A) ERF of CO_2_. (B) ERF of CH_4_. (C) ERF of N_2_O. (D) ERF of aerosols. (E) ERF of contrails.

This study compares the changes in CO_2_, CH_4_, N_2_O, contrails, and aerosol ERF from 2023 to 2100 under the four scenarios of only ETS (scenario 1), only SAFs (scenario 2), the hydrogen turbine engines mixed with SAFs (scenario 3) and the hydrogen fuel cells mixed with SAFs (scenario 4) with the baseline scenario. Figure 3 shows the ERF changes of CO_2_, CH_4_, N_2_O, contrails, and aerosol under five scenarios from 2023 to 2100.

And the control of CH_4_ ERF, N_2_O ERF, and contrails ERF under the only ETS scenarios is not significant, so it is also not shown in the figure. From Figures 3A and 2C, compared to the baseline scenario, the sub-scenarios with the most significant ERF changes and lowest ERF changes under the five scenarios are consistent with the sub-scenarios under the concentration changes indicator, and the ERF trends of CO_2_, CH_4_, and N_2_O are also roughly the same.

Figure 3A shows the ERF changes of CO_2_ from 2023 to 2100. In the case of the baseline scenario, compared with 2022, the growth of CO_2_ ERF will be linearly and incrementally by 3.59E-07 W/m^2^ by 2100. For scenario 1, scenario 2, scenario 3, and scenario 4, their sub-scenario with the smallest change in ERF changes of CO_2_ (scenario 1-2 (2040–2055), scenario 2-1 (2025-50%), scenario 3, and scenario 4-3) also show a significant decrease compared to the baseline scenario. And the most significant performance is in the nine scenarios of the hydrogen turbine engines mixed with SAFs (scenario 3). In scenario 3-4, by 2100, the minimum CO_2_ ERF is only 7.58E-08 W/m^2^, approximately 21.12% of the baseline scenario. Under nine scenarios of the hydrogen fuel cells mixed with SAFs (scenario 4), by 2100, the minimum CO_2_ ERF is 7.99 E−08 W/m^2^, not significantly different from scenario 3 overall. Next are the only ETS scenarios (scenario 1) and the only SAF scenario (scenario 2). The minimum CO_2_ ERF is 9.37E-08 W/m^2^ and 1.22E-07 W/m^2^, respectively. Figure 3B shows the ERF changes of CH_4_ from 2023 to 2100. As shown in Figure 3B, by 2100, the minimum CH_4_ ERF is only 6.68E-07 W/m^2^. Next are the only SAF scenarios. By 2100, the minimum ERF change of CH_4_ is 1.06E-06 W/m^2^. As for the changes of ERF of N_2_O, it can be seen from Figure 3C that the minimum N_2_O ERF of scenario 2, scenario 3, and scenario 4 are 8.98E-06 W/m^2^ (scenario 2-2), 1.01E-05 W/m^2^ (scenario 3-6) and 8.98E-06 W/m^2^ (scenario 4-4), respectively, by 2100. Moreover, compared with CO_2_ ERF and CH_4_ ERF, the control effect of scenario 3 on N_2_O ERF is not as good as that of scenario 4.

Figure 3D shows the ERF changes of aerosols from 2023 to 2100. The ERF of aerosols increases under scenario 1, scenario 2, scenario 3, and scenario 4. In general, the effective forced radiation of aerosols is negative. However, the aerosol ERF values of scenario 1, scenario 2, scenario 3, and scenario 4 are higher than those of the baseline scenario. The ERF of aerosols will be −5.48E-11 W/m^2^ by 2100 (scenario 1-1 and scenario 1-2), with the slightest overall change in the scenario with only ETS. Next is the scenario with only SAF. By 2100, the ERF of aerosols will be −5.96E-11 W/m^2^ under 2025-50% (scenario 2-1). However, under the two scenarios of 2025-50% + 2030-100% and 2025-50% + 2035-100%, the ERF of aerosols by 2100 is positive, both of which are 8.12E-13 W/m^2^. Finally, in scenario 3 and scenario 4, in the sub-scenarios with the slightest change in aerosol ERF, the aerosol ERF will be −3.94E-11 W/m^2^ (scenario 3-3), −3.54E-11 W/m^2^ (scenario 4-3) by 2100. And the aerosol ERF is positive in the sub-scenarios with the most considerable change.

As for the changes in the ERF of contrails, by 2100, the best overall control of contrails’ ERF is scenario 4, scenario 3, and scenario 2. However, compared with the baseline scenario, by 2100, the best sub-scenarios (scenario 2-2, scenario 3-6, and scenario 4-4) of scenario 2, scenario 3, and scenario 4 can make the ERF of contrails to be 0 W/m^2^.

Although aviation carbon emissions are growing rapidly and must be strictly controlled, the ERF caused by CO_2_ is insignificant. However, other aviation emissions (such as N_2_O and CH_4_) have greater ERF and may still cause an inevitable temperature rise. If no measures are taken, the ERF growth of N_2_O is the highest, followed by CH_4_ by 2100. Therefore, the measures to reduce other emissions should also be given high attention, just like carbon reduction measures.

Although the control effect of only the ETS scenario on CO_2_ concentration and ERF is better than that of only the SAF scenario, the control effect of the ETS scenario on CH_4_ concentration and ERF, N_2_O concentration and ERF and contrails ERF is not as good as that of SAF.

And under the combined scenario of hydrogen turbine engines and SAF, compared with the scenario of only SAF, it can be found that the use of hydrogen turbine engines can further affect the CO_2_ concentration and ERF, N_2_O concentration, and its ERF, CH_4_ concentration, and its ERF, aerosol, and contrails ERF, for example, For CO_2_ concentration and ERF, compared with using only SAF, the use of hydrogen turbine engines can further reduce it by about 13%. But, compared with the scenario where only SAF is used, the combination of hydrogen fuel cells and SAF can reduce the CO_2_ concentration and ERF increase by about 12%, which shows that the effect is not as good as that of hydrogen turbine engines.

### Temperature changes in the five scenarios of global aviation

**Figure 4:**
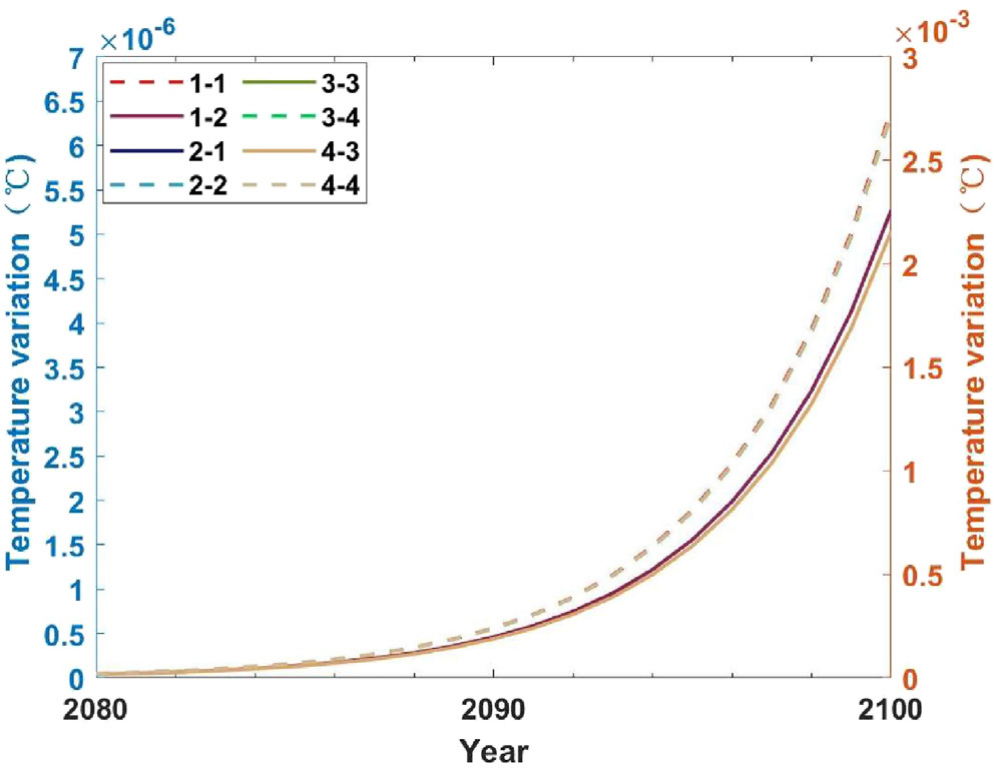
Temperature changes under five scenarios from 2023 to 2100 (difference from baseline scenario)

As for temperature changes, the difference in temperature changes between 2023 and 2080 is not significant, so Figure 4 shows the temperature changes between 2080 and 2100 under scenario 1, scenario 2, scenario 3, and scenario 4. The left axis represents the two sub-scenarios of scenario 2, while the right axis represents the others.

By 2100, compared with the baseline scenario, the rise in temperature appreciation under the four scenarios decreased. The control effect on temperature in descending order is the only ETS scenario (scenario 1), the only SAF scenario (scenario 2), the hydrogen fuel cells mixed with SAFs (scenario 4), and the hydrogen turbine engines mixed with SAFs (scenario 3). Under the three scenarios of SAF, the temperature rise will decrease by 2.16E-03°C–2.72E-03°C (scenario 2-1 and scenario 2-2) by 2100, while under the scenario with only carbon trading scheme scenario (scenario 1), the maximum decrease value is 6.39E-06°C (scenario 1-2). Therefore, using SAF is more effective in controlling the pollutant emissions of the route, thus reducing the impact on the climate more significantly. In the nine cases of scenario 3, the most significant decrease in temperature rise by 2100 will be 2.72E-03°C (scenario 3-4), which has less impact on climate compared with the carbon trading scheme (the lowest rising value is 6.39E-06°C). Compared with the scenario in which only SAF is used, the overall temperature change has little difference. Still, the decrease in temperature rise in scenario 3-4 is 2.72E-03°C, higher than the three scenarios in which only SAF is used. Compared with SAF and hydrogen turbine engines, the temperature change has little difference.

## Discussion

This study focuses on the impact of global aviation pollutant emissions on climate change. First, this study collects the CO_2_, CO, HC, NO_x_, PM2.5, and SO_2_ emissions from global routes to better summarize the impact of aircraft activities on the environment and provide data and method references for proposing corresponding countermeasures. Then, the FAIR method is systematically designed for aviation emissions. The climate effects of global routes under different scenarios are calculated: the baseline scenario, the scenario with only carbon emission trading schemes, the scenario with only SAF, the scenario with a hydrogen turbine engine and SAF, the scenario with HFC and SAF. Discuss the concentration changes of greenhouse gases (carbon dioxide, methane, and nitrous oxide), ERF (including aviation aerosols and contrails), and temperature changes from 2023 to 2100.

The main contributions of this study to the literature are reflected in the following aspects. First, this study attempts to establish a systematic framework to study the impact of air pollutant emissions on climate change. Currently, the FAIR method is widely used to calculate the atmospheric concentration of greenhouse gases and the ERF of greenhouse gases, aerosols, ozone, and other driving factors. Still, it has not been specially designed and applied to aviation, and this research can fill this gap. Secondly, this study attempts to compare and analyze the two main ways of aviation emission reduction-carbon emission trading schemes and SAF. Some papers focus on studying carbon emission trading schemes or SAF, but no study can compare the two. In addition, based on the application scenario of biomass fuel, this study considers the impact of the combination of hydrogen turbines and hydrogen fuel cells with biomass fuel on climate change. Unfortunately, there is little relevant literature on the systematic study of using hydrogen energy (liquid hydrogen) in the aviation industry.

The main conclusions and policy recommendations are as follows.

First, by comparing the concentration changes of CO_2_, CH_4_, and N_2_O, this study can find that the impact of CO_2_ on the greenhouse effect remains the greatest. If not controlled, in the baseline scenario, by 2100, the concentration will increase by 1.86E-05 ppm, ranking second in N_2_O concentration.

Second, the four scenarios of only ETS (scenario 1), only SAFs (scenario 2), the hydrogen turbine engines mixed with SAFs (scenario 3), and the hydrogen fuel cells mixed with SAFs (scenario 4) have better control over carbon dioxide concentration and ERF. The most significant performance is in the nine scenarios of hydrogen turbine engines. In scenario 3-4, by 2100, the carbon dioxide concentration is approximately 21.12% of the unconstrained scenario. The second is the hydrogen fuel cells mixed with SAFs (scenario 4), and the last is only the ETS and SAF scenarios.

Third, scenario 3 and scenario 4 are still the two most significant scenarios for controlling CH_4_ concentration and ERF. By 2100, the minimum CH_4_ concentration was only 2.41E-10ppb, approximately 12% of the baseline scenario. Finally, there is the SAF scenario.

Fourth, unlike CO_2_ and CH_4_, the hydrogen turbine engines mixed with SAFs (scenario 3) has less control over N_2_O concentration and ERF than the hydrogen fuel cells mixed with SAFs (scenario 4). Under scenario 4, the minimum N_2_O concentration and ERF by 2100 are only 6.12E-09ppb and 8.98E-06 W/m^2^.

Fifth, the ERF of aerosols is negative. However, the aerosol ERF values of scenario 1, scenario 2, scenario 3, and scenario 4 are higher than those of the baseline scenario. In the only ETS scenario, the overall change is relatively minimal, and by 2100, the ERF of aerosols will reach −5.48E-11 W/m^2^. The ERF of aerosols in scenario 2, scenario 3, and scenario 4 may all be greater than 0 W/m^2^.

Sixth, by 2100, the best overall control of contrails' ERF is scenario 4, scenario 3, and scenario 2. And compared with the baseline scenario, by 2100, the best sub-scenarios (scenario 2-2, scenario 3-6, and scenario 4-4) of scenario 2, scenario 3, and scenario 4 can make the ERF of contrails to be 0 W/m^2^.

Seventh, although the control effect of only the ETS scenario on CO_2_ concentration and ERF is better than that of only the SAF scenario, the control effect of the ETS scenario on CH_4_ concentration and ERF, N_2_O concentration and ERF and contrails ERF is not as good as that of SAF.

Eighth, under the combined scenario of hydrogen turbine engines and SAF, compared with the scenario of only SAF, it can be found that the use of hydrogen turbine engines can further affect the CO_2_ concentration and ERF, N_2_O concentration and its ERF, CH_4_ concentration, and its ERF, aerosol, and contrails ERF, for example, For CO_2_ concentration and ERF, compared with using only SAF, the use of hydrogen turbine engines can further reduce it by about 13%. But, compared with the scenario where only SAF is used, the combination of hydrogen fuel cells and SAF can reduce the CO_2_ concentration and ERF increase by about 12%, which shows that the effect is not as good as that of hydrogen turbine engines. This result may be due to limitations such as technology and deployment speed.

Ninth, by 2100, compared to the baseline scenario, the temperature increase in the four scenarios has decreased. Under the three scenarios of SAF, by 2100, the temperature rise will decrease by 2.16E-03°C and 2.72E-03°C (scenario 2-1 and scenario 2-2, respectively), while under the scenario of only carbon emission trading scenario (scenario 1), the maximum decrease is 6.39E-06°C (scenario 1-2). Therefore, SAFs can more effectively control airline pollutant emissions, significantly reducing their impact on climate. The overall difference in temperature control between scenario 3 and scenario 4 is insignificant, but both are better than scenarios that only use SAF and ETS. Among them, scenario 3-4 is the most significant, with a decrease in temperature rise of 2.72E-03°C, which is higher than the decrease in other scenarios.

This study has several limitations. First, although this study analyzed the possible future emissions under the scenarios of using hydrogen energy and SAFs, it does not compare and calculate the cost of hydrogen energy and the usage costs of biomass fuels. Therefore, it is necessary to conduct further research based on the data in this study, such as cost analysis and control, to explore the feasibility of using hydrogen energy and biomass fuels for future aviation and provide valuable suggestions for formulating suitable plans for sustainable development, energy conservation, and emission reduction. In addition, this study has not considered the application of carbon capture technology. Therefore, the following research direction can calculate the cost of using hydrogen energy for feasibility analysis.

## STAR★Methods

### Key resources table

**Table:** 

REAGENT or RESOURCE	SOURCE	IDENTIFIER
**Deposited data**		

Carbon emissions of global aviation industry	IATA annual review (2023)	https://www.iata.org/en/publications/annual-review/
Proportion of four body aircrafts	Oliverwyman (2023)	https://www.oliverwyman.com/content/dam/oliverwyman/v2/publications/2022/feb/MRO-2022-Master-file_v5.pdf

### Resource availability

#### Lead contact

Further information and requests should be directed to the lead author, Qiang Cui (cuiqiang@seu.edu.cn).

#### Materials availability

This study did not generate new unique materials.

#### Data and code availability

• The overall emissions of global aviation industry during 2015–-2021 can be found in Table S1.^38^ The concentration changes of CO_2_, CH_4_, and N_2_O, the ERF changes, and temperature changes under the different scenarios are shown in Tables S1, S2, S3, S4, S5, S6, S7, S8, S9, and S10.^38^
• The results are calculated through MATLAB R2014b, and the codes are shown in the supplementary information.
• Any additional information required to reanalyze the data reported in this paper is available from the lead contact upon request.

### Method details

#### Method to calculate the annual emissions during 2015–2021

The data on carbon emissions of global aviation industry from 2015 to 2021 is from the IATA annual review,^39^ which were 774,000,000 tons, 812,000,000 tons, 860,000,000 tons, 905,000,000 tons, 915,000,000 tons, 646,000,000 tons and 726,000,000 tons. However, there are no existing data on the other emissions.

In general, global aircraft can be divided into wide body aircraft, narrow body aircraft, regional aircraft, and turboprop aircraft. This study collects the proportion of these four body types from the data of oliverwyman^40^ and calculate the average emission coefficient per ton of aviation kerosene through the Modified BFFM2-FOA-FPM method.^41^^,^^42^ The average proportions of these four body types during 2015–2021 were 59.56% (narrow body), 12.80% (regional aircraft), 18.92% (wide body), and 8.72% (turboprop).^40^ Therefore, this study gets the emission coefficients of per ton of aviation kerosene through the Modified BFFM2-FOA-FPM method, which are 3.87 g/kg (SO_2_), 2.494 g/kg (HC), 25.548 g/kg (NO_x_), and 0.292 g/kg (PM2.5). Based on these data, this study gets the detailed emissions of the other pollutions, as shown in Table S1.

#### Aviation-FAIR (Aviation - Finite Amplitude Impulse Response)

Aircraft emissions are CO_2_, CO, HC, NO_x_, SO_2_, and PM2.5.^41^^,^^42^ The primary greenhouse gases are CO_2_, CH_4_ (part of HC), and N_2_O (part of NO_x_), the calculation of contrails’ ERF is related to NO_x_,^43^^,^^44^^,^^45^ and aerosols are related to SO_2_, NO_x_, and PM2.5.^46^ Based on the FAIR 1.3 model^46^ and the reality of the aviation industry, this study builds a general framework for Aviation-FAIR.

According to the FAIR method,^46^ CO_2_ would be partitioned into four boxes: geological processes ($\tau_{0}$), the deep ocean ($\tau_{1}$), the biosphere ($\tau_{2}$), and the ocean mixed layer ($\tau_{3}$). The partition fractions are $\alpha_{i}$ and $\sum_{i=0}^{3}\alpha_{i}=1$. The concentration of CO_2_ is

(Equation 1) $$C_{{CO}_{2}}=278+\sum_{i=0}^{3}\frac{R_{i}}{M_{a}}\frac{\omega_{{CO}_{2}}}{\omega }$$

$M_{a}=5.1352*10^{18}\;\text{kg}$ is the dry mass of the atmosphere, $\omega_{{CO}_{2}}=44.01$ is the molecular weight of CO_2_, and ω=28.966 is the molecular weight of dry air.

In Equation 1, the formula of $R_{i}$ is

(Equation 2) $$\frac{dR_{i}}{dt}=\alpha_{i}E_{{CO}_{2}}-\frac{R_{i}}{\partial \tau_{i}},i=0,1,2,3$$

$E_{{CO}_{2}}$ is the CO_2_ emission.

The variable ∂ is gotten by

(Equation 3) $$\sum_{i=0}^{3}\partial \alpha_{i}\tau_{i}\left[1-\exp\;\left(\frac{-100}{{\partial \tau }_{i}}\right)\right]=r_{0}+r_{C}\left[\sum_{t}E_{{CO}_{2},t}-C_{{CO}_{2}}+278\right]+r_{T}\Delta T$$

$r_{c}=0.019$ yt GtC^-1^, $r_{T}=4.165$ years K^-1^, and $r_{0}=35$. ΔT is the temperature change.

The (Equation 1), (Equation 2), (Equation 3) form a cycle, and this study use one hundred thousand Monte Carlo simulations to solve them.

As part of CH_4_ and NO_x_ (the proportion of CH_4_ in aviation HC is 0.4 and that of N_2_O in aviation NO_x_ is 0.29),^47^^,^^48^ for CH_4_ and N_2_O, their concentrations are

(Equation 4) $$C_{t}=C_{t-1}+\frac{1}{2}\left(\delta C_{t-1}+\delta C_{t}\right)-C_{t-1}\left(1-\exp\left(-\frac{1}{\tau }\right)\right)$$

τ the atmospheric lifetime. For CH_4_, τ=9.3; For N_2_O, τ=121.

$\delta C_{t}$ can be gotten by

(Equation 5) $$\delta C_{t}=\frac{E_{t}}{M_{a}}\frac{\omega }{\omega_{f}}\text{.}$$

$\omega_{f}$ is the molecular mass. For CH_4_, $\omega_{f}=16.04$; For N_2_O, $\omega_{f}=44.01$. $E_{t}$ is CH_4_ or N_2_O emission of year t.

The Effective Radiative Forcing of CO_2_, N_2_O and CH_4_ are

(Equation 6) $$F_{{CO}_{2}}=\left[\left(-2.4\times 10^{-7}\right){\left(C_{{CO}_{2}}-C_{{CO}_{2}pi}\right)}^{2}+\left(7.2\times 10^{-4}\right)\left|C_{{CO}_{2}}-C_{{CO}_{2}pi}\right|-\left(1.05\times 10^{-4}\right)\left(C_{N2O}+C_{N_{2}Opi}\right)+5.36\right]\times \log\left(\frac{C_{{CO}_{2}}}{C_{{CO}_{2}pi}}\right)\text{.}$$

(Equation 7) $$F_{N_{2}O}=\left[\left(-4.0\times 10^{-6}\right){\left(C_{{CO}_{2}}+C_{{CO}_{2}pi}\right)}^{2}+\left(2.1\times 10^{-6}\right)\left(C_{N_{2}O}+C_{N_{2}Opi}\right)-\left(2.45\times 10^{-6}\right)\left(C_{{CH}_{4}}+C_{{CH}_{4}pi}\right)+0.117\right]\times \left(\sqrt{C_{N_{2}O}}-\sqrt{C_{N_{2}Opi}}\right)\text{.}$$

(Equation 8) $$F_{{CH}_{4}}=\left[\left(-6.5\times 10^{-7}\right){\left(C_{{CH}_{4}}+C_{{CH}_{4}pi}\right)}^{2}-\left(4.1\times 10^{-6}\right)\left(C_{N_{2}O}+C_{N_{2}Opi}\right)+0.043\right]\times \left(\sqrt{C_{{CH}_{4}}}-\sqrt{C_{{CH}_{4}pi}}\right)\text{.}$$

$C_{{CO}_{2}pi}$, $C_{{CH}_{4}pi}$, $C_{N_{2}Opi}$ are the benchmark concentration of CO_2_, N_2_O and CH_4_. Because focusing on the ERF change, this study sets them as the concentration for the year 2022.

The ERF of Contrails is

(Equation 9) $$F_{con}=0.0152\times \frac{E_{NOx}}{M_{a}}\text{.}$$

The ERF of aerosols is

(Equation 10) $$F_{aer}=\frac{\left(0.08\times E_{{PM}_{2.5}}-0.34\times E_{{SO}_{2}}-0.044\times E_{NOx}\right)}{M_{a}}\text{.}$$

$E_{{PM}_{2.5}}$, $E_{{SO}_{2}}$, and $E_{NOx}$are the emissions of PM2.5, SO_2_ and NO_x_.

The temperature change is

(Equation 11) $${\Delta T}_{t}=\Delta T_{t,1}\;\exp\left(\frac{1}{d_{1}}\right)+\left(1-\exp\left(\frac{1}{d_{1}}\right)\right)\times q_{1}\times \left(F_{{CO}_{2}}+F_{N_{2}O}+F_{{CH}_{4}}+F_{con}+F_{aer}\right)+\Delta T_{t,2}\;\exp\left(\frac{1}{d_{2}}\right)+\left(1-\exp\left(\frac{1}{d_{2}}\right)\right)\times q_{2}\times \left(F_{{CO}_{2}}+F_{N_{2}O}+F_{{CH}_{4}}+F_{con}+F_{aer}\right)\text{.}$$

$d_{1}=239$ and $d_{2}=4.1$ indicate the responses to forcing from the upper ocean and the deep ocean. $q_{1}$ and $q_{2}$ are the coefficients, and in this study, $q_{1}=3.8729E-04$, $q_{2}=-3.836E-04$.

### Quantification and statistical analysis

This study does not include statistical analysis or quantification.

## Supplemental information

Document S1. Supplementary information for “Path analysis for controlling climate change in global aviation ”

Table S1. The emissions of six pollutions from 2015 to 2022, related to STAR Methods

Table S2. The concentration of CO2 from 2023 to 2100, related to STAR Methods

Table S3. The concentration of CH4 from 2023 to 2100, related to STAR Methods

Table S4. The concentration of N2O from 2023 to 2100, related to STAR Methods

Table S5. The effective radiative forcing of CO2 from 2023 to 2100, related to STAR Methods

Table S6. The effective radiative forcing of CH4 from 2023 to 2100, related to STAR Methods

Table S7. The effective radiative forcing of N2O from 2023 to 2100, related to STAR Methods

Table S8. The effective radiative forcing of aerosols from 2023 to 2100, related to STAR Methods

Table S9. The effective radiative forcing of contrails from 2023 to 2100, related to STAR Methods

Table S10. The temperature change from 2023 to 2100, related to STAR Methods
