# Supplementary material for: Path analysis for controlling climate change in global aviation
Source: iScience. 2024 May 28;27(6):110126. doi: 10.1016/j.isci.2024.110126 (PMC11214314; doi:10.1016/j.isci.2024.110126)
Supplement: Document S1. Supplementary information for “Path analysis for controlling climate change in global aviation — ” [file mmc1.pdf]

iScience, Volume *27*

Supplemental information

Path analysis for controlling climate change in global aviation

Qiang Cui, Yi-lin Lei, Zi-ke Jia, Yu Wang, and Ye Li

Supplementary Information for "Path Analysis for Controlling Climate Change in Global Aviation"

Qiang Cui [1,4,5], Yi-lin Lei[1], Zi-ke Jia[1], Yu Wang[2,4], Ye Li[3,4,5]

[1]School of Economics and Management, Southeast University, Nanjing, China

[2]School of Economics and Management, Civil Aviation Flight University of China, Guanghan, China

[3]School of Business Administration, Nanjing University of Finance and Economics, Nanjing, China

[4]These authors contributed equally.

[5]Lead contact: Correspondence: cuiqiang@seu.edu.cn (Qiang Cui); happyxiaoye@yeah.net (Ye Li).

This supplementary information contains two parts: annual emissions under the scenarios, and detailed MATLAB codes.

1. Annual emissions under the scenarios

This paper has set four scenarios: the baseline scenario (Scenario 0), the scenario of only Emissions Trading Scheme (ETS) (Scenario 1), the scenario of only Sustainable Aviation Fuels (SAFs) (Scenario 2) and the scenario of hydrogen energy mixed with SAFs. The last one contains two sub-scenarios: hydrogen turbine engine mixed with SAFs (Scenario 3) and hydrogen fuel cell mixed with SAFs (Scenario 4).

For the baseline scenario (Scenario 0), this study assumes that the annual pollutant emissions after 2022 are the average of 2015-2021. The International Air Transport Association (IATA) committed in 2021 that emissions from global aviation would be net-zero by 2050 [1]. However, the aviation industry is still developing, and its annual emissions are likely to exceed 2019 for a period. They may decline later due to emission constraints and alternative energy. Similarly, the global aviation emissions have been increasing from 2015 to 2019, but the emissions will decrease in 2020 and 2021 due to the impact of the COVID-19. This process is somewhat like the trend of future global aviation emissions, so it is reasonable to set the average emissions from 2015 to 2021 as the baseline scenario.

Therefore, the annual emissions of CO_2, HC, NOx, PM2.5, and SO_2 are 805,428,571.4 tons, 636,280.9 tons, 6,517,924.97 tons, 74,496.4 tons, and 987,332.46 tons.

For the scenario of only ETS (Scenario 1), two sub- scenarios are set: Scenario 1-1 and Scenario 1-2. According to the goal of International Air Transport Association (IATA), if the aviation carbon peak can be achieved by 2035, it is scenario 1-1, 2035-2050. The current carbon emissions will continue until 2035, and the zero-carbon will be achieved by 2050[2]. If current carbon emissions will continue until 2040, the scenario is 1-2, 2040-2055. The zero-carbon will be achieved in 2055. In this scenario, the CO_2 and SO_2 emissions (the SO_2 changes with the change of CO_2) will change but the other emissions remain unchanged.

Therefore, for Scenario 1-1, CO_2 and SO_2 emissions remain 805,428,571.4 tons and 987,332.46 tons until 2035 and will decrease annually to lead to zero emissions by 2050. For Scenario 1-2, CO_2 and SO_2 emissions remain 805,428,571.4 tons and 987,332.46 tons until 2040 and will decrease annually to lead to zero emissions by 2055.

For the scenario of only SAFs (Scenario 2), three sub-scenarios are set: 2025-50% (Scenario 2-1), 2025-50%+2030-100% (Scenario 2-2), and 2025-50%+2035-100% (Scenario 2-3). Compared with kerosene fuel, the PM2.5 produced by SAFs can be reduced by 30%, and the carbon dioxide

and methane can be reduced by 50%-90%, and no NOx and SO_2 will produced by SAFs. This study assumes that CO_2 and HC will reduce by 70% (the average value) when the SAFs are used. By 2022, the maximum mixing proportion of sustainable aviation fuel will be only 50%[2]. Still, Airbus has tested 100% of sustainable aviation fuel on mainstream aircraft and is expected to achieve this in 2030[2,3].

Under Scenario 2-1, the emissions before 2025 are the average value of 2015-2021, and from 2025, CO_2 and HC will become (1-0.5*0.7)* Scenario 0, NOx and SO_2 will become 0.5* Scenario 0 and PM 2.5 will become (1-0.5*0.3)* Scenario 0.

Under Scenario 2-2, the emissions before 2025 are the average value of 2015-2021, and from 2025 to 2029, CO_2 and HC will become (1-0.5*0.7)* Scenario 0, NOx and SO_2 will become 0.5* Scenario 0 and PM2.5 will become (1-0.5*0.3)* Scenario 0. Then from 2030, CO_2 and HC will become (1-0.7)* Scenario 0, NOx and SO_2 will become 0* Scenario 0 and PM2.5 will become (1-0.3)* Scenario 0.

Under Scenario 2-3, the emissions before 2025 are the average value of 2015-2021, and from 2025 to 2034, CO_2 and HC will become (1-0.5*0.7)* Scenario 0, NOx and SO_2 will become 0.5* Scenario 0 and PM 2.5 will become (1-0.5*0.3)* Scenario 0. Then after 2035, CO_2 and HC will become (1-0.7)* Scenario 0, NOx and SO_2 will become 0* Scenario 0 and PM2.5 will become (1-0.3)* Scenario 0.

For the scenario of Hydrogen Turbine engines (HT) mixed with SAFs (Scenario 3), nine sub-scenarios are set: 3-1: 2025&$SAF_{50\%}$+2035&$HT_{20\%}$&$SAF_{50\%}$+2045&$HT_{40\%}$&$SAF_{50\%}$+2055&$HT_{60\%}$&$SAF_{40\%}$; 3-2: 2025&$SAF_{50\%}$+2035&$HT_{0\%}$&$SAF_{50\%}$+2045&$HT_{20\%}$&$SAF_{50\%}$+2055&$HT_{40\%}$&$SAF_{50\%}$; 3-3: 2025&$SAF_{50\%}$+2035&$HT_{0\%}$&$SAF_{50\%}$+2045&$HT_{0\%}$&$SAF_{50\%}$+2055&$HT_{20\%}$&$SAF_{50\%}$; 3-4: 2025&$SAF_{50\%}$+2030&$SAF_{100\%}$+2035&$HT_{20\%}$&$SAF_{80\%}$+2045&$HT_{40\%}$&$SAF_{60\%}$+2055&$HT_{60\%}$&$SAF_{40\%}$; 3-5: 2025&$SAF_{50\%}$+2030&$SAF_{100\%}$+2035&$HT_{0\%}$&$SAF_{100\%}$+2045&$HT_{20\%}$&$SAF_{80\%}$+2055&$HT_{40\%}$&$SAF_{60\%}$; 3-6: 2025&$SAF_{50\%}$+2030&$SAF_{100\%}$+2035&$HT_{0\%}$&$SAF_{100\%}$+2045&$HT_{0\%}$&$SAF_{100\%}$+2055&$HT_{20\%}$&$SAF_{80\%}$; 3-7: 2025&$SAF_{50\%}$+2035&$HT_{20\%}$&$SAF_{80\%}$+2045&$HT_{40\%}$&$SAF_{60\%}$+2055&$HT_{60\%}$&$SAF_{40\%}$; 3-8: 2025&$SAF_{50\%}$+2035&$HT_{0\%}$&$SAF_{100\%}$+2045&$HT_{20\%}$&$SAF_{80\%}$+2055&$HT_{40\%}$&$SAF_{60\%}$; 3-9: 2025&$SAF_{50\%}$+2035&$HT_{0\%}$&$SAF_{100\%}$+2045&$HT_{0\%}$&$SAF_{100\%}$+2055&$HT_{20\%}$&$SAF_{80\%}$.

According to Airbus's plan, the hydrogen turbine engine is expected to be commercially available in 2035[4]. Still, considering the deployment efficiency of less developed countries such as Africa, the commercial proportion will not be too large.

For example, Scenario 3-1 shows that 50% of sustainable aviation fuel will be used in 2025, 20% of hydrogen turbine engines and 50% of sustainable aviation fuel will be used in 2035, 40% of hydrogen turbine engines and 50% of sustainable aviation fuel will be used in 2045, and 60% of hydrogen turbine engine and 40% of sustainable aviation fuel will be used in 2055.

Compared with ordinary engines, the application of hydrogen turbine engines will reduce NO_x by 50%-80%. At the same time, CO_2 emission is 0%, HC is 0%, and PM2.5 are reduced by 30-50%. This study assumes that NO_x will reduce by 65% (the average value) and the PM2.5 will reduce by 40% when the hydrogen turbine engines are used[6].

Under Scenario 3-1, the emissions before 2025 are the average value of 2015-2021, and from

2025 to 2034, CO_2 and HC will become (1-0.5*0.7)* Scenario 0, NOx and SO_2 will become 0.5* Scenario 0 and PM2.5 will become (1-0.5*0.3)* Scenario 0. Then from 2035 to 2044, CO_2 and HC will become (1-0.7*0.5-0.2)* Scenario 0, NOx and SO_2 will become (1-0.5-0.2*0.65)* Scenario 0 and PM2.5 will become (1-0.3*0.5-0.2*0.4)* Scenario 0. From 2045 to 2054, CO_2 and HC will become (1-0.7*0.5-0.4)* Scenario 0, NOx and SO_2 will become (1-0.5-0.4*0.65)* Scenario 0 and PM2.5 will become (1-0.3*0.5-0.4*0.4)* Scenario 0. After 2055, CO_2 and HC will become (1-0.7*0.5-0.6)* Scenario 0, NOx and SO_2 will become (1-0.5-0.6*0.65)* Scenario 0 and PM2.5 will become (1-0.3*0.5-0.6*0.4)* Scenario 0.

Under Scenario 3-2, the emissions before 2025 are the average value of 2015-2021, and from 2025 to 2034, CO_2 and HC will become (1-0.5*0.7)* Scenario 0, NOx and SO_2 will become 0.5* Scenario 0 and PM2.5 will become (1-0.5*0.3)* Scenario 0. Then from 2035 to 2044, CO_2 and HC will become (1-0.7*0.5-0.2)* Scenario 0, NOx and SO_2 will become (1-0.5-0.2*0.65)* Scenario 0 and PM2.5 will become (1-0.3*0.5-0.2*0.4)* Scenario 0. From 2045 to 2054, CO_2 and HC will become (1-0.7*0.5-0.2)* Scenario 0, NOx and SO_2 will become (1-0.5-0.2*0.65)* Scenario 0 and PM2.5 will become (1-0.3*0.5-0.2*0.4)* Scenario 0. After 2055, CO_2 and HC will become (1-0.7*0.5-0.4)* Scenario 0, NOx and SO_2 will become (1-0.5-0.4*0.65)* Scenario 0 and PM2.5 will become (1-0.3*0.5-0.4*0.4)* Scenario 0.

Under Scenario 3-3, the emissions before 2025 are the average value of 2015-2021, and from 2025 to 2054, CO_2 and HC will become (1-0.5*0.7)* Scenario 0, NOx and SO_2 will become 0.5* Scenario 0 and PM2.5 will become (1-0.5*0.3)* Scenario 0. After 2055, CO_2 and HC will become (1-0.7*0.5-0.2)* Scenario 0, NOx and SO_2 will become (1-0.5-0.2*0.65)* Scenario 0 and PM2.5 will become (1-0.3*0.5-0.2*0.4)* Scenario 0.

Under Scenario 3-4, the emissions before 2025 are the average value of 2015-2021, and from 2025 to 2029, CO_2 and HC will become (1-0.5*0.7)* Scenario 0, NOx and SO_2 will become 0.5* Scenario 0 and PM2.5 will become (1-0.5*0.3)* Scenario 0. From 2030 to 2034, CO_2 and HC will become (1-0.7)* Scenario 0, NOx and SO_2 will become 0* Scenario 0 and PM2.5 will become (1-0.3)* Scenario 0. Then from 2035 to 2044, CO_2 and HC will become (1-0.7*0.8-0.2)* Scenario 0, NOx and SO_2 will become (1-0.8-0.2*0.65)* Scenario 0 and PM2.5 will become (1-0.3*0.8-0.2*0.4)* Scenario 0. From 2045 to 2054, CO_2 and HC will become (1-0.7*0.6-0.4)* Scenario 0, NOx and SO_2 will become (1-0.6-0.4*0.65)* Scenario 0 and PM2.5 will become (1-0.3*0.6-0.4*0.4)* Scenario 0. After 2055, CO_2 and HC will become (1-0.7*0.4-0.6)* Scenario 0, NOx and SO_2 will become (1-0.4-0.6*0.65)* Scenario 0 and PM2.5 will become (1-0.3*0.4-0.4*0.6)* Scenario 0.

Under Scenario 3-5, the emissions before 2025 are the average value of 2015-2021, and from 2025 to 2029, CO_2 and HC will become (1-0.5*0.7)* Scenario 0, NOx and SO_2 will become 0.5* Scenario 0 and PM2.5 will become (1-0.5*0.3)* Scenario 0. From 2030 to 2044, CO_2 and HC will become (1-0.7)* Scenario 0, NOx and SO_2 will become 0* Scenario 0 and PM2.5 will become (1-0.3)* Scenario 0. From 2045 to 2054, CO_2 and HC will become (1-0.7*0.8-0.2)* Scenario 0, NOx and SO_2 will become (1-0.8-0.2*0.65)* Scenario 0 and PM2.5 will become (1-0.3*0.8-0.2*0.4)* Scenario 0. After 2055, CO_2 and HC will become (1-0.7*0.6-0.4)* Scenario 0, NOx and SO_2 will become (1-0.6-0.4*0.65)* Scenario 0 and PM2.5 will become (1-0.3*0.6-0.4*0.4)* Scenario 0.

Under Scenario 3-6, the emissions before 2025 are the average value of 2015-2021, and from 2025 to 2029, CO_2 and HC will become (1-0.5*0.7)* Scenario 0, NOx and SO_2 will become 0.5* Scenario 0 and PM2.5 will become (1-0.5*0.3)* Scenario 0. From 2030 to 2054, CO_2 and HC will

become (1-0.7)* Scenario 0, NOx and SO_2 will become 0* Scenario 0 and PM2.5 will become (1-0.3)* Scenario 0. After 2055, CO_2 and HC will become (1-0.7*0.8-0.2)* Scenario 0, NOx and SO_2 will become (1-0.8-0.2*0.65)* Scenario 0 and PM2.5 will become (1-0.3*0.8-0.2*0.4)* Scenario 0.

Under Scenario 3-7, the emissions before 2025 are the average value of 2015-2021, and from 2025 to 2034, CO_2 and HC will become (1-0.5*0.7)* Scenario 0, NOx and SO_2 will become 0.5* Scenario 0 and PM2.5 will become (1-0.5*0.3)* Scenario 0. From 2035 to 2044, CO_2 and HC will become (1-0.7*0.8-0.2)* Scenario 0, NOx and SO_2 will become (1-0.8-0.2*0.65)* Scenario 0 and PM2.5 will become (1-0.3*0.8-0.2*0.4)* Scenario 0. From 2045 to 2054, CO_2 and HC will become (1-0.7*0.6-0.4)* Scenario 0, NOx and SO_2 will become (1-0.6-0.4*0.65)* Scenario 0 and PM2.5 will become (1-0.3*0.6-0.4*0.4)* Scenario 0. After 2055, CO_2 and HC will become (1-0.7*0.4-0.6)* Scenario 0, NOx and SO_2 will become (1-0.4-0.6*0.65)* Scenario 0 and PM2.5 will become (1-0.3*0.4-0.6*0.4)* Scenario 0.

Under Scenario 3-8, the emissions before 2025 are the average value of 2015-2021, and from 2025 to 2034, CO_2 and HC will become (1-0.5*0.7)* Scenario 0, NOx and SO_2 will become 0.5* Scenario 0 and PM2.5 will become (1-0.5*0.3)* Scenario 0. From 2035 to 2044, CO_2 and HC will become (1-0.7)* Scenario 0, NOx and SO_2 will become 0* Scenario 0 and PM2.5 will become (1-0.3)* Scenario 0. From 2045 to 2054, CO_2 and HC will become (1-0.7*0.8-0.2)* Scenario 0, NOx and SO_2 will become (1-0.8-0.2*0.65)* Scenario 0 and PM2.5 will become (1-0.3*0.8-0.2*0.4)* Scenario 0. After 2055, CO_2 and HC will become (1-0.7*0.6-0.4)* Scenario 0, NOx and SO_2 will become (1-0.6-0.4*0.65)* Scenario 0 and PM2.5 will become (1-0.3*0.6-0.4*0.4)* Scenario 0.

Under Scenario 3-9, the emissions before 2025 are the average value of 2015-2021, and from 2025 to 2034, CO_2 and HC will become (1-0.5*0.7)* Scenario 0, NOx and SO_2 will become 0.5* Scenario 0 and PM2.5 will become (1-0.5*0.3)* Scenario 0. From 2035 to 2054, CO_2 and HC will become (1-0.7)* Scenario 0, NOx and SO_2 will become 0* Scenario 0 and PM2.5 will become (1-0.3)* Scenario 0. After 2055, CO_2 and HC will become (1-0.7*0.8-0.2)* Scenario 0, NOx and SO_2 will become (1-0.8-0.2*0.65)* Scenario 0 and PM2.5 will become (1-0.3*0.8-0.2*0.4)* Scenario 0.

For the scenario of Hydrogen Fuel Cells (HFC) mixed with SAFs (Scenario 4), nine sub-scenarios are set: 4-1: 2025&$SAF_{50\%}$+2040&$HFC_{20\%}$&$SAF_{50\%}$+2050&$HFC_{40\%}$&$SAF_{50\%}$+2060&$HFC_{60\%}$ &$SAF_{40\%}$; 4-2:2025&$SAF_{50\%}$+2040&$HFC_{0\%}$&$SAF_{50\%}$+2050&$HFC_{20\%}$&$SAF_{50\%}$+2060&$HFC_{40\%}$ &$SAF_{50\%}$; 4-3:2025&$SAF_{50\%}$+2040&$HFC_{0\%}$&$SAF_{50\%}$+2050&$HFC_{0\%}$&$SAF_{50\%}$+2060&$HFC_{20\%}$ &$SAF_{50\%}$; 4-4:2025&$SAF_{50\%}$+2030&$SAF_{100\%}$+2040&$HFC_{20\%}$&$SAF_{80\%}$+2050&$HFC_{40\%}$ &$SAF_{60\%}$+2060&$HFC_{60\%}$ &$SAF_{40\%}$; 4-5:2025&$SAF_{50\%}$+2030&$SAF_{100\%}$+2040&$HFC_{0\%}$&$SAF_{100\%}$+2050&$HFC_{20\%}$ &$SAF_{80\%}$+2060&$HFC_{40\%}$ &$SAF_{60\%}$; 4-6:2025&$SAF_{50\%}$+2030&$SAF_{100\%}$+2040&$HFC_{0\%}$&$SAF_{100\%}$+2050&$HFC_{0\%}$ &$SAF_{100\%}$+2060&$HFC_{20\%}$ &$SAF_{80\%}$; 4-7:2025&$SAF_{50\%}$+2040&$HFC_{20\%}$&$SAF_{80\%}$+2050&$HFC_{40\%}$&$SAF_{60\%}$+2060&$HFC_{60\%}$ &$SAF_{40\%}$; 4-8:2025&$SAF_{50\%}$+2040&$HFC_{0\%}$&$SAF_{100\%}$+2050&$HFC_{20\%}$&$SAF_{80\%}$+2060&$HFC_{40\%}$ &$SAF_{60\%}$; 4-9:2025&$SAF_{50\%}$+2040&$HFC_{0\%}$&$SAF_{100\%}$+2050&$HFC_{0\%}$&$SAF_{100\%}$+2060&$HFC_{20\%}$ &$SAF_{80\%}$.

For example, Scenario 4-1 shows that 50% of sustainable aviation fuel will be used in 2025, 20% of hydrogen fuel cells and 50% of sustainable aviation fuel will be used in 2040, 40% of hydrogen fuel cells and 50% of sustainable aviation fuel will be used in 2050, and 60% of hydrogen fuel cells and 40% of sustainable aviation fuel will be used in 2055.

Compared with ordinary engines, hydrogen fuel cells only produce water, and CO_2, HC, and NO_x emissions are 0%, which can reduce PM2.5 by 60%-80%. This study assumes that PM2.5 will reduce by 70% when hydrogen fuel cells are used[5].

Under Scenario 4-1, the emissions before 2025 are the average value of 2015-2021, and from 2025 to 2039, CO_2 and HC will become (1-0.5*0.7)* Scenario 0, NOx and SO_2 will become 0.5* Scenario 0 and PM2.5 will become (1-0.5*0.3)* Scenario 0. Then from 2040 to 2049, CO_2 and HC will become (1-0.7*0.5-0.2)* Scenario 0, the NOx and SO_2 will become (1-0.5-0.2)* Scenario 0 and PM2.5 will become (1-0.3*0.5-0.2*0.7)* Scenario 0. From 2050 to 2059, the CO_2 and HC will become (1-0.7*0.5-0.4)* Scenario 0, NOx and SO_2 will become (1-0.5-0.4)* Scenario 0 and PM2.5 will become (1-0.3*0.5-0.4*0.7)* Scenario 0. After 2060, CO_2 and HC will become (1-0.7*0.4-0.6)* Scenario 0, NOx and SO_2 will become (1-0.4-0.6)* Scenario 0 and the PM 2.5 will become (1-0.3*0.4-0.6*0.7)* Scenario 0.

Under Scenario 4-2, the emissions before 2025 are the average value of 2015-2021, and from 2025 to 2049, CO_2 and HC will become (1-0.5*0.7)* Scenario 0, NOx and SO_2 will become 0.5* Scenario 0 and PM2.5 will become (1-0.5*0.3)* Scenario 0. Then from 2050 to 2059, CO_2 and HC will become (1-0.7*0.5-0.2)* Scenario 0, NOx and SO_2 will become (1-0.5-0.2)* Scenario 0 and PM2.5 will become (1-0.3*0.5-0.2*0.7)* Scenario 0. After 2060, CO_2 and HC will become (1-0.7*0.5-0.4)* Scenario 0, the NOx and SO_2 will become (1-0.5-0.4)* Scenario 0 and the PM 2.5 will become (1-0.3*0.5-0.4*0.7)* Scenario 0.

Under Scenario 4-3, the emissions before 2025 are the average value of 2015-2021, and from 2025 to 2059, CO_2 and HC will become (1-0.5*0.7)* Scenario 0, NOx and SO_2 will become 0.5* Scenario 0 and PM2.5 will become (1-0.5*0.3)* Scenario 0. After 2060, CO_2 and HC will become (1-0.7*0.5-0.2)* Scenario 0, NOx and SO_2 will become (1-0.5-0.2)* Scenario 0 and PM2.5 will become (1-0.3*0.5-0.2*0.7)* Scenario 0.

Under Scenario 4-4, the emissions before 2025 are the average value of 2015-2021, and from 2025 to 2029, CO_2 and HC will become (1-0.5*0.7)* Scenario 0, NOx and SO_2 will become 0.5* Scenario 0 and PM2.5 will become (1-0.5*0.3)* Scenario 0. From 2030 to 2039, CO_2 and HC will become (1-0.7)* Scenario 0, NOx and SO_2 will become 0* Scenario 0 and PM2.5 will become (1-0.3)* Scenario 0. From 2040 to 2049, CO_2 and HC will become (1-0.7*0.8-0.2)* Scenario 0, NOx and SO_2 will become (1-0.8-0.2)* Scenario 0 and PM2.5 will become (1-0.3*0.8-0.2*0.7)* Scenario 0. From 2050 to 2059, CO_2 and HC will become (1-0.7*0.6-0.4)* Scenario 0, NOx and SO_2 will become (1-0.6-0.4)* Scenario 0 and PM2.5 will become (1-0.3*0.6-0.4*0.7)* Scenario 0. After 2060, CO_2 and HC will become (1-0.7*0.4-0.6)* Scenario 0, NOx and SO_2 will become (1-0.4-0.6)* Scenario 0 and PM2.5 will become (1-0.3*0.4-0.6*0.7)* Scenario 0.

Under Scenario 4-5, the emissions before 2025 are the average value of 2015-2021, and from 2025 to 2029, CO_2 and HC will become (1-0.5*0.7)* Scenario 0, NOx and SO_2 will become 0.5* Scenario 0 and PM2.5 will become (1-0.5*0.3)* Scenario 0. From 2030 to 2049, CO_2 and HC will become (1-0.7)* Scenario 0, NOx and SO_2 will become 0* Scenario 0 and PM2.5 will become (1-0.3)* Scenario 0. From 2050 to 2059, CO_2 and HC will become (1-0.7*0.8-0.2)* Scenario 0, NOx and SO_2 will become (1-0.8-0.2)* Scenario 0 and PM2.5 will become (1-0.3*0.8-0.2*0.7)* Scenario 0. After 2060, CO_2 and HC will become (1-0.7*0.6-0.4)* Scenario 0, NOx and SO_2 will become (1-0.6-0.4)* Scenario 0 and PM2.5 will become (1-0.3*0.6-0.4*0.7)* Scenario 0.

Under Scenario 4-6, the emissions before 2025 are the average value of 2015-2021, and from 2025 to 2029, CO_2 and HC will become (1-0.5*0.7)* Scenario 0, NOx and SO_2 will become 0.5*

Scenario 0 and PM2.5 will become (1-0.5*0.3)* Scenario 0. From 2030 to 2059, CO_2 and HC will become (1-0.7)* Scenario 0, NOx and SO_2 will become 0* Scenario 0 and PM2.5 will become (1-0.3)* Scenario 0. After 2060, CO_2 and HC will become (1-0.7*0.6-0.4)* Scenario 0, NOx and SO_2 will become (1-0.6-0.4)* Scenario 0 and PM2.5 will become (1-0.3*0.6-0.4*0.7)* Scenario 0.

Under Scenario 4-7, the emissions before 2025 are the average value of 2015-2021, and from 2025 to 2039, CO_2 and HC will become (1-0.5*0.7)* Scenario 0, NOx and SO_2 will become 0.5* Scenario 0 and PM2.5 will become (1-0.5*0.3)* Scenario 0. From 2040 to 2049, CO_2 and HC will become (1-0.7*0.8-0.2)* Scenario 0, NOx and SO_2 will become (1-0.8-0.2)* Scenario 0 and PM2.5 will become (1-0.3*0.8-0.2*0.7)* Scenario 0. From 2050 to 2059, CO_2 and HC will become (1-0.7*0.6-0.4)* Scenario 0, NOx and SO_2 will become (1-0.6-0.4)* Scenario 0 and PM2.5 will become (1-0.3*0.6-0.4*0.7)* Scenario 0. After 2060, CO_2 and HC will become (1-0.7*0.4-0.6)* Scenario 0, NOx and SO_2 will become (1-0.4-0.6)* Scenario 0 and he PM2.5 will become (1-0.3*0.4-0.6*0.7)* Scenario 0.

Under Scenario 4-8, the emissions before 2025 are the average value of 2015-2021, and from 2025 to 2039, CO_2 and HC will become (1-0.5*0.7)* Scenario 0, NOx and SO_2 will become 0.5* Scenario 0 and PM2.5 will become (1-0.5*0.3)* Scenario 0. From 2040 to 2049, CO_2 and HC will become (1-0.7)* Scenario 0, NOx and SO_2 will become 0* Scenario 0 and PM2.5 will become (1-0.3)* Scenario 0. From 2050 to 2059, CO_2 and HC will become (1-0.7*0.8-0.2)* Scenario 0, NOx and SO_2 will become (1-0.8-0.2)* Scenario 0 and PM2.5 will become (1-0.3*0.8-0.2*0.7)* Scenario 0. After 2060, CO_2 and HC will become (1-0.7*0.6-0.4)* Scenario 0, NOx and SO_2 will become (1-0.6-0.4)* Scenario 0 and PM2.5 will become (1-0.3*0.6-0.4*0.7)* Scenario 0.

Under Scenario 4-9, the emissions before 2025 are the average value of 2015-2021, and from 2025 to 2039, CO_2 and HC will become (1-0.5*0.7)* Scenario 0, NOx and SO_2 will become 0.5* Scenario 0 and PM2.5 will become (1-0.5*0.3)* Scenario 0. From 2040 to 2059, CO_2 and HC will become (1-0.7)* Scenario 0, NOx and SO_2 will become 0* Scenario 0 and PM2.5 will become (1-0.3)* Scenario 0. After 2060, CO_2 and HC will become (1-0.7*0.8-0.2)* Scenario 0, NOx and SO_2 will become (1-0.8-0.2)* Scenario 0 and PM2.5 will become (1-0.3*0.8-0.2*0.7)* Scenario 0.

2. Detailed MATLAB codes

The results are calculated through MATLAB R2014b, the detailed codes are as follows.

```
% Scenario 0
%The annual emissions, and this study assumes the period is 300 years
%The first year is 2023
ECO20=10^3*805428571.428571*ones(1,300);% The annual emissions of CO2(kg)
EHC=10^3*636280.917688583*ones(1,300); % The annual emissions of HC
ENOx=10^3*6517924.97398072*ones(1,300); % The annual emissions of NOx
EPM=10^3*74496.4025521517*ones(1,300); % The annual emissions of PM2.5
ESO2=10^3*987332.458482284*ones(1,300); % The annual emissions of SO2
% Scenario 1-1
ECO20=10^3*[805428571.4   805428571.4 805428571.4 805428571.4 805428571.4 805428571.4
805428571.4 805428571.4 805428571.4 805428571.4 805428571.4 805428571.4 805428571.4
841270142.9 785185466.7 729100790.5 673016114.3 616931438.1 560846761.9 504762085.7
448677409.5 392592733.3 336508057.1 280423381   224338704.8 168254028.6 112169352.4
56084676.19 0   0   0   0   0   0   0   0   0   0*ones(1,262)];
```

```
EHC=10^3*ones(1,300)*636280.9177;
ENOx=10^3*ones(1,300)*6517924.974;
EPM=10^3*ones(1,300)*74496.40255;
ESO2=10^3*[987332.4585 987332.4585 987332.4585 987332.4585 987332.4585 987332.4585
987332.4585 987332.4585 987332.4585 987332.4585 987332.4585 987332.4585 987332.4585
1031268.753 962517.5027 893766.2525 825015.0023 756263.7521 687512.5019 618761.2517
550010.0015 481258.7513 412507.5012 343756.251  275005.0008 206253.7506 137502.5004
68751.25019 0   0   0   0   0   0   0   0   0   0   0*ones(1,262)];

% Scenario 1-2
%ECO20=10^3*[805428571.4 805428571.4 805428571.4 805428571.4 805428571.4 805428571.4
805428571.4 805428571.4 805428571.4 805428571.4 805428571.4 805428571.4 805428571.4
805428571.4 805428571.4 805428571.4 805428571.4 805428571.4 841270142.9 785185466.7
729100790.5 673016114.3 616931438.1 560846761.9 504762085.7 448677409.5 392592733.3
336508057.1 280423381   224338704.8 168254028.6 112169352.4 56084676.19 0   0   0   0   0
0*ones(1,262)];
%EHC=10^3*ones(1,300)*636280.9177;
%ENOx=10^3*ones(1,300)*6517924.974;
%EPM=10^3*ones(1,300)*74496.40255;
%ESO2=10^3*[987332.4585  987332.4585 987332.4585 987332.4585 987332.4585 987332.4585
987332.4585 987332.4585 987332.4585 987332.4585 987332.4585 987332.4585 987332.4585
987332.4585 987332.4585 987332.4585 987332.4585 987332.4585 1031268.753 962517.5027
893766.2525 825015.0023 756263.7521 687512.5019 618761.2517 550010.0015 481258.7513
412507.5012 343756.251  275005.0008 206253.7506 137502.5004 68751.25019 0   0   0   0   0
0*ones(1,262)];

% Scenario 2-1
%ECO20=10^3*[805428571.4   805428571.4 805428571.4 523528571.4 523528571.4 523528571.4
523528571.4 523528571.4 523528571.4 523528571.4 523528571.4 523528571.4 523528571.4
523528571.4 523528571.4 523528571.4 523528571.4 523528571.4 523528571.4 523528571.4
523528571.4 523528571.4 523528571.4 523528571.4 523528571.4 523528571.4 523528571.4
523528571.4 523528571.4 523528571.4 523528571.4 523528571.4 523528571.4 523528571.4
523528571.4 523528571.4 523528571.4 523528571.4 523528571.4*ones(1,262)];
%EHC=10^3*[636280.9177  636280.9177 636280.9177 413582.5965 413582.5965 413582.5965
413582.5965 413582.5965 413582.5965 413582.5965 413582.5965 413582.5965 413582.5965
413582.5965 413582.5965 413582.5965 413582.5965 413582.5965 413582.5965 413582.5965
413582.5965 413582.5965 413582.5965 413582.5965 413582.5965 413582.5965 413582.5965
413582.5965 413582.5965 413582.5965 413582.5965 413582.5965 413582.5965 413582.5965
413582.5965 413582.5965 413582.5965 413582.5965 413582.5965*ones(1,262)];
%ENOx=10^3*[6517924.974 6517924.974 6517924.974 3258962.487 3258962.487 3258962.487
3258962.487 3258962.487 3258962.487 3258962.487 3258962.487 3258962.487 3258962.487
3258962.487 3258962.487 3258962.487 3258962.487 3258962.487 3258962.487 3258962.487
3258962.487 3258962.487 3258962.487 3258962.487 3258962.487 3258962.487 3258962.487
3258962.487 3258962.487 3258962.487 3258962.487 3258962.487 3258962.487 3258962.487
```

```
3258962.487 3258962.487 3258962.487 3258962.487 3258962.487*ones(1,262)];
%EPM=10^3*[74496.40255  74496.40255 74496.40255 63321.94217 63321.94217 63321.94217
63321.94217 63321.94217 63321.94217 63321.94217 63321.94217 63321.94217 63321.94217
63321.94217 63321.94217 63321.94217 63321.94217 63321.94217 63321.94217 63321.94217
63321.94217 63321.94217 63321.94217 63321.94217 63321.94217 63321.94217 63321.94217
63321.94217 63321.94217 63321.94217 63321.94217 63321.94217 63321.94217 63321.94217
63321.94217 63321.94217 63321.94217 63321.94217 63321.94217*ones(1,262)];
%ESO2=10^3*[987332.4585 987332.4585 987332.4585 493666.2292 493666.2292 493666.2292
493666.2292 493666.2292 493666.2292 493666.2292 493666.2292 493666.2292 493666.2292
493666.2292 493666.2292 493666.2292 493666.2292 493666.2292 493666.2292 493666.2292
493666.2292 493666.2292 493666.2292 493666.2292 493666.2292 493666.2292 493666.2292
493666.2292 493666.2292 493666.2292 493666.2292 493666.2292 493666.2292 493666.2292
493666.2292 493666.2292 493666.2292 493666.2292 493666.2292*ones(1,262)];

% Scenario 2-2
%ECO20=10^3*[805428571.4   805428571.4 805428571.4 523528571.4 523528571.4 523528571.4
523528571.4 523528571.4 241628571.4 241628571.4 241628571.4 241628571.4 241628571.4
241628571.4 241628571.4 241628571.4 241628571.4 241628571.4 241628571.4 241628571.4
241628571.4 241628571.4 241628571.4 241628571.4 241628571.4 241628571.4 241628571.4
241628571.4 241628571.4 241628571.4 241628571.4 241628571.4 241628571.4 241628571.4
241628571.4 241628571.4 241628571.4 241628571.4 241628571.4*ones(1,262)];
%EHC=10^3*[636280.9177  636280.9177 636280.9177 413582.5965 413582.5965 413582.5965
413582.5965 413582.5965 190884.2753 190884.2753 190884.2753 190884.2753 190884.2753
190884.2753 190884.2753 190884.2753 190884.2753 190884.2753 190884.2753 190884.2753
190884.2753 190884.2753 190884.2753 190884.2753 190884.2753 190884.2753 190884.2753
190884.2753 190884.2753 190884.2753 190884.2753 190884.2753 190884.2753 190884.2753
190884.2753 190884.2753 190884.2753 190884.2753 190884.2753*ones(1,262)];
%ENOx=10^3*[6517924.974 6517924.974 6517924.974 3258962.487 3258962.487 3258962.487
3258962.487 3258962.487 0   0   0   0   0   0   0   0   0   0   0   0   0   0   0   0   0   0   0   0
0   0   0   0   0   0   0   0   0   0   0*ones(1,262)];
%EPM=10^3*[74496.40255  74496.40255 74496.40255 63321.94217 63321.94217 63321.94217
63321.94217 63321.94217 52147.48179 52147.48179 52147.48179 52147.48179 52147.48179
52147.48179 52147.48179 52147.48179 52147.48179 52147.48179 52147.48179 52147.48179
52147.48179 52147.48179 52147.48179 52147.48179 52147.48179 52147.48179 52147.48179
52147.48179 52147.48179 52147.48179 52147.48179 52147.48179 52147.48179 52147.48179
52147.48179 52147.48179 52147.48179 52147.48179 52147.48179*ones(1,262)];
%ESO2=10^3*[987332.4585 987332.4585 987332.4585 493666.2292 493666.2292 493666.2292
493666.2292 493666.2292 0   0   0   0   0   0   0   0   0   0   0   0   0   0   0   0   0   0   0   0
0   0   0   0   0   0   0   0   0   0   0*ones(1,262)];

% Scenario 2-3
ECO20=10^3*[805428571.4 805428571.4 805428571.4 523528571.4 523528571.4 523528571.4
523528571.4 523528571.4 523528571.4 523528571.4 523528571.4 523528571.4 523528571.4
241628571.4 241628571.4 241628571.4 241628571.4 241628571.4 241628571.4 241628571.4
```

```
241628571.4 241628571.4 241628571.4 241628571.4 241628571.4 241628571.4 241628571.4
241628571.4 241628571.4 241628571.4 241628571.4 241628571.4 241628571.4 241628571.4
241628571.4 241628571.4 241628571.4 241628571.4 241628571.4*ones(1,262)];
EHC=10^3*[636280.9177  636280.9177 636280.9177 413582.5965 413582.5965 413582.5965
413582.5965 413582.5965 413582.5965 413582.5965 413582.5965 413582.5965 413582.5965
190884.2753 190884.2753 190884.2753 190884.2753 190884.2753 190884.2753 190884.2753
190884.2753 190884.2753 190884.2753 190884.2753 190884.2753 190884.2753 190884.2753
190884.2753 190884.2753 190884.2753 190884.2753 190884.2753 190884.2753 190884.2753
190884.2753 190884.2753 190884.2753 190884.2753 190884.2753*ones(1,262)];
ENOx=10^3*[6517924.974  6517924.974 6517924.974 3258962.487 3258962.487 3258962.487
3258962.487 3258962.487 3258962.487 3258962.487 3258962.487 3258962.487 3258962.487 0   0   0
0   0   0   0   0   0   0   0   0   0   0   0   0   0   0   0   0   0   0   0   0   0   0*ones(1,262)];
EPM=10^3*[74496.40255  74496.40255 74496.40255 63321.94217 63321.94217 63321.94217
63321.94217 63321.94217 63321.94217 63321.94217 63321.94217 63321.94217 63321.94217
52147.48179 52147.48179 52147.48179 52147.48179 52147.48179 52147.48179 52147.48179
52147.48179 52147.48179 52147.48179 52147.48179 52147.48179 52147.48179 52147.48179
52147.48179 52147.48179 52147.48179 52147.48179 52147.48179 52147.48179 52147.48179
52147.48179 52147.48179 52147.48179 52147.48179 52147.48179*ones(1,262)];
ESO2=10^3*[987332.4585  987332.4585 987332.4585 493666.2292 493666.2292 493666.2292
493666.2292 493666.2292 493666.2292 493666.2292 493666.2292 493666.2292 493666.2292 0   0   0
0   0   0   0   0   0   0   0   0   0   0   0   0   0   0   0   0   0   0   0   0   0   0*ones(1,262)];

% Scenario 3-1
%ECO20=10^3*[805428571.4   805428571.4 805428571.4 523528571.4 523528571.4 523528571.4
523528571.4 523528571.4 523528571.4 523528571.4 523528571.4 523528571.4 523528571.4
362442857.1 362442857.1 362442857.1 362442857.1 362442857.1 362442857.1 362442857.1
362442857.1 362442857.1 362442857.1 201357142.9 201357142.9 201357142.9 201357142.9
201357142.9 201357142.9 201357142.9 201357142.9 201357142.9 201357142.9 96651428.57
96651428.57 96651428.57 96651428.57 96651428.57 96651428.57*ones(1,262)];
%EHC=10^3*[636280.9177  636280.9177 636280.9177 413582.5965 413582.5965 413582.5965
413582.5965 413582.5965 413582.5965 413582.5965 413582.5965 413582.5965 413582.5965
286326.413  286326.413  286326.413  286326.413  286326.413  286326.413  286326.413  286326.413
286326.413  286326.413  159070.2294 159070.2294 159070.2294 159070.2294 159070.2294
159070.2294 159070.2294 159070.2294 159070.2294 159070.2294 76353.71012 76353.71012
76353.71012 76353.71012 76353.71012 76353.71012*ones(1,262)];
%ENOx=10^3*[6517924.974 6517924.974 6517924.974 3258962.487 3258962.487 3258962.487
3258962.487 3258962.487 3258962.487 3258962.487 3258962.487 3258962.487 3258962.487
2411632.24  2411632.24  2411632.24  2411632.24  2411632.24  2411632.24  2411632.24  2411632.24
2411632.24  2411632.24  1564301.994 1564301.994 1564301.994 1564301.994 1564301.994
1564301.994 1564301.994 1564301.994 1564301.994 1564301.994 1368764.245 1368764.245
1368764.245 1368764.245 1368764.245 1368764.245*ones(1,262)];
%EPM=10^3*[74496.40255  74496.40255 74496.40255 63321.94217 63321.94217 63321.94217
63321.94217 63321.94217 63321.94217 63321.94217 63321.94217 63321.94217 63321.94217
57362.22997 57362.22997 57362.22997 57362.22997 57362.22997 57362.22997 57362.22997
```

```
57362.22997 57362.22997 57362.22997 51402.51776 51402.51776 51402.51776 51402.51776
51402.51776 51402.51776 51402.51776 51402.51776 51402.51776 51402.51776 47677.69763
47677.69763 47677.69763 47677.69763 47677.69763 47677.69763*ones(1,262)];
%ESO2=10^3*[987332.4585 987332.4585 987332.4585 493666.2292 493666.2292 493666.2292
493666.2292 493666.2292 493666.2292 493666.2292 493666.2292 493666.2292 493666.2292
296199.7375 296199.7375 296199.7375 296199.7375 296199.7375 296199.7375 296199.7375
296199.7375 296199.7375 296199.7375 98733.24585 98733.24585 98733.24585 98733.24585
98733.24585 98733.24585 98733.24585 98733.24585 98733.24585 98733.24585 0   0   0   0   0
0*ones(1,262)];

% Scenario 3-2
%ECO20=10^3*[805428571.4   805428571.4 805428571.4 523528571.4 523528571.4 523528571.4
523528571.4 523528571.4 523528571.4 523528571.4 523528571.4 523528571.4 523528571.4
523528571.4 523528571.4 523528571.4 523528571.4 523528571.4 523528571.4 523528571.4
523528571.4 523528571.4 523528571.4 362442857.1 362442857.1 362442857.1 362442857.1
362442857.1 362442857.1 362442857.1 362442857.1 362442857.1 362442857.1 201357142.9
201357142.9 201357142.9 201357142.9 201357142.9 201357142.9*ones(1,262)];
%EHC=10^3*[636280.9177  636280.9177 636280.9177 413582.5965 413582.5965 413582.5965
413582.5965 413582.5965 413582.5965 413582.5965 413582.5965 413582.5965 413582.5965
413582.5965 413582.5965 413582.5965 413582.5965 413582.5965 413582.5965 413582.5965
413582.5965 413582.5965 413582.5965 286326.413  286326.413  286326.413  286326.413
286326.413  286326.413  286326.413  286326.413  286326.413  286326.413  159070.2294
159070.2294 159070.2294 159070.2294 159070.2294 159070.2294*ones(1,262)];
%ENOx=10^3*[6517924.974 6517924.974 6517924.974 3258962.487 3258962.487 3258962.487
3258962.487 3258962.487 3258962.487 3258962.487 3258962.487 3258962.487 3258962.487
3258962.487 3258962.487 3258962.487 3258962.487 3258962.487 3258962.487 3258962.487
3258962.487 3258962.487 3258962.487 2411632.24  2411632.24  2411632.24  2411632.24
2411632.24  2411632.24  2411632.24  2411632.24  2411632.24  2411632.24  1564301.994
1564301.994 1564301.994 1564301.994 1564301.994 1564301.994*ones(1,262)];
%EPM=10^3*[74496.40255  74496.40255 74496.40255 63321.94217 63321.94217 63321.94217
63321.94217 63321.94217 63321.94217 63321.94217 63321.94217 63321.94217 63321.94217
63321.94217 63321.94217 63321.94217 63321.94217 63321.94217 63321.94217 63321.94217
63321.94217 63321.94217 63321.94217 57362.22997 57362.22997 57362.22997 57362.22997
57362.22997 57362.22997 57362.22997 57362.22997 57362.22997 57362.22997 51402.51776
51402.51776 51402.51776 51402.51776 51402.51776 51402.51776*ones(1,262)];
%ESO2=10^3*[987332.4585 987332.4585 987332.4585 493666.2292 493666.2292 493666.2292
493666.2292 493666.2292 493666.2292 493666.2292 493666.2292 493666.2292 493666.2292
493666.2292 493666.2292 493666.2292 493666.2292 493666.2292 493666.2292 493666.2292
493666.2292 493666.2292 493666.2292 296199.7375 296199.7375 296199.7375 296199.7375
296199.7375 296199.7375 296199.7375 296199.7375 296199.7375 296199.7375 98733.24585
98733.24585 98733.24585 98733.24585 98733.24585 98733.24585*ones(1,262)];


% Scenario 3-3
```

```
%ECO20=10^3*[805428571.4   805428571.4 805428571.4 523528571.4 523528571.4 523528571.4
523528571.4 523528571.4 523528571.4 523528571.4 523528571.4 523528571.4 523528571.4
523528571.4 523528571.4 523528571.4 523528571.4 523528571.4 523528571.4 523528571.4
523528571.4 523528571.4 523528571.4 523528571.4 523528571.4 523528571.4 523528571.4
523528571.4 523528571.4 523528571.4 523528571.4 523528571.4 523528571.4 362442857.1
362442857.1 362442857.1 362442857.1 362442857.1 362442857.1*ones(1,262)];
%EHC=10^3*[636280.9177  636280.9177 636280.9177 413582.5965 413582.5965 413582.5965
413582.5965 413582.5965 413582.5965 413582.5965 413582.5965 413582.5965 413582.5965
413582.5965 413582.5965 413582.5965 413582.5965 413582.5965 413582.5965 413582.5965
413582.5965 413582.5965 413582.5965 413582.5965 413582.5965 413582.5965 413582.5965
413582.5965 413582.5965 413582.5965 413582.5965 413582.5965 413582.5965 286326.413
286326.413  286326.413  286326.413  286326.413  286326.413*ones(1,262)];
%ENOx=10^3*[6517924.974 6517924.974 6517924.974 3258962.487 3258962.487 3258962.487
3258962.487 3258962.487 3258962.487 3258962.487 3258962.487 3258962.487 3258962.487
3258962.487 3258962.487 3258962.487 3258962.487 3258962.487 3258962.487 3258962.487
3258962.487 3258962.487 3258962.487 3258962.487 3258962.487 3258962.487 3258962.487
3258962.487 3258962.487 3258962.487 3258962.487 3258962.487 3258962.487 2411632.24
2411632.24  2411632.24  2411632.24  2411632.24  2411632.24*ones(1,262)];
%EPM=10^3*[74496.40255  74496.40255 74496.40255 63321.94217 63321.94217 63321.94217
63321.94217 63321.94217 63321.94217 63321.94217 63321.94217 63321.94217 63321.94217
63321.94217 63321.94217 63321.94217 63321.94217 63321.94217 63321.94217 63321.94217
63321.94217 63321.94217 63321.94217 63321.94217 63321.94217 63321.94217 63321.94217
63321.94217 63321.94217 63321.94217 63321.94217 63321.94217 63321.94217 57362.22997
57362.22997 57362.22997 57362.22997 57362.22997 57362.22997*ones(1,262)];
%ESO2=10^3*[987332.4585 987332.4585 987332.4585 493666.2292 493666.2292 493666.2292
493666.2292 493666.2292 493666.2292 493666.2292 493666.2292 493666.2292 493666.2292
493666.2292 493666.2292 493666.2292 493666.2292 493666.2292 493666.2292 493666.2292
493666.2292 493666.2292 493666.2292 493666.2292 493666.2292 493666.2292 493666.2292
493666.2292 493666.2292 493666.2292 493666.2292 493666.2292 493666.2292 296199.7375
296199.7375 296199.7375 296199.7375 296199.7375 296199.7375*ones(1,262)];

% Scenario 3-4
%ECO20=10^3*[805428571.4   805428571.4 805428571.4 523528571.4 523528571.4 523528571.4
523528571.4 523528571.4 241628571.4 241628571.4 241628571.4 241628571.4 241628571.4
193302857.1 193302857.1 193302857.1 193302857.1 193302857.1 193302857.1 193302857.1
193302857.1 193302857.1 193302857.1 144977142.9 144977142.9 144977142.9 144977142.9
144977142.9 144977142.9 144977142.9 144977142.9 144977142.9 144977142.9 96651428.57
96651428.57 96651428.57 96651428.57 96651428.57 96651428.57*ones(1,262)];
%EHC=10^3*[636280.9177  636280.9177 636280.9177 413582.5965 413582.5965 413582.5965
413582.5965 413582.5965 190884.2753 190884.2753 190884.2753 190884.2753 190884.2753
152707.4202 152707.4202 152707.4202 152707.4202 152707.4202 152707.4202 152707.4202
152707.4202 152707.4202 152707.4202 114530.5652 114530.5652 114530.5652 114530.5652
114530.5652 114530.5652 114530.5652 114530.5652 114530.5652 114530.5652 76353.71012
76353.71012 76353.71012 76353.71012 76353.71012 76353.71012*ones(1,262)];
```

```
%ENOx=10^3*[6517924.974 6517924.974 6517924.974 3258962.487 3258962.487 3258962.487
3258962.487 3258962.487 0   0   0   0   0   456254.7482 456254.7482 456254.7482 456254.7482
456254.7482 456254.7482 456254.7482 456254.7482 456254.7482 456254.7482 912509.4964
912509.4964 912509.4964 912509.4964 912509.4964 912509.4964 912509.4964 912509.4964
912509.4964 912509.4964 1368764.245 1368764.245 1368764.245 1368764.245 1368764.245
1368764.245*ones(1,262)];
%EPM=10^3*[74496.40255  74496.40255 74496.40255 63321.94217 63321.94217 63321.94217
63321.94217 63321.94217 52147.48179 52147.48179 52147.48179 52147.48179 52147.48179
50657.55374 50657.55374 50657.55374 50657.55374 50657.55374 50657.55374 50657.55374
50657.55374 50657.55374 50657.55374 49167.62568 49167.62568 49167.62568 49167.62568
49167.62568 49167.62568 49167.62568 49167.62568 49167.62568 49167.62568 47677.69763
47677.69763 47677.69763 47677.69763 47677.69763 47677.69763*ones(1,262)];
%ESO2=10^3*[987332.4585 987332.4585 987332.4585 493666.2292 493666.2292 493666.2292
493666.2292 493666.2292 0   0   0   0   0   0   0   0   0   0   0   0   0   0   0   0   0   0   0   0
0   0   0   0   0   0   0   0   0   0   0*ones(1,262)];

% Scenario 3-5
%ECO20=10^3*[805428571.4   805428571.4 805428571.4 523528571.4 523528571.4 523528571.4
523528571.4 523528571.4 523528571.4 523528571.4 523528571.4 523528571.4 523528571.4
523528571.4 523528571.4 523528571.4 523528571.4 523528571.4 523528571.4 523528571.4
523528571.4 523528571.4 523528571.4 193302857.1 193302857.1 193302857.1 193302857.1
193302857.1 193302857.1 193302857.1 193302857.1 193302857.1 193302857.1 144977142.9
144977142.9 144977142.9 144977142.9 144977142.9 144977142.9*ones(1,262)];
%EHC=10^3*[636280.9177  636280.9177 636280.9177 413582.5965 413582.5965 413582.5965
413582.5965 413582.5965 413582.5965 413582.5965 413582.5965 413582.5965 413582.5965
413582.5965 413582.5965 413582.5965 413582.5965 413582.5965 413582.5965 413582.5965
413582.5965 413582.5965 413582.5965 152707.4202 152707.4202 152707.4202 152707.4202
152707.4202 152707.4202 152707.4202 152707.4202 152707.4202 152707.4202 114530.5652
114530.5652 114530.5652 114530.5652 114530.5652 114530.5652*ones(1,262)];
%ENOx=10^3*[6517924.974 6517924.974 6517924.974 3258962.487 3258962.487 3258962.487
3258962.487 3258962.487 0   0   0   0   0   0   0   0   0   0   0   0   0   0   0   456254.7482
456254.7482 456254.7482 456254.7482 456254.7482 456254.7482 456254.7482 456254.7482
456254.7482 456254.7482 912509.4964 912509.4964 912509.4964 912509.4964 912509.4964
912509.4964*ones(1,262)];
%EPM=10^3*[74496.40255  74496.40255 74496.40255 63321.94217 63321.94217 63321.94217
63321.94217 63321.94217 52147.48179 52147.48179 52147.48179 52147.48179 52147.48179
52147.48179 52147.48179 52147.48179 52147.48179 52147.48179 52147.48179 52147.48179
52147.48179 52147.48179 52147.48179 50657.55374 50657.55374 50657.55374 50657.55374
50657.55374 50657.55374 50657.55374 50657.55374 50657.55374 50657.55374 49167.62568
49167.62568 49167.62568 49167.62568 49167.62568 49167.62568*ones(1,262)];
%ESO2=10^3*[987332.4585 987332.4585 987332.4585 493666.2292 493666.2292 493666.2292
493666.2292 493666.2292 0   0   0   0   0   0   0   0   0   0   0   0   0   0   0   0   0   0   0   0
0   0   0   0   0   0   0   0   0   0   0*ones(1,262)];
```

```
% Scenario 3-6
%ECO20=10^3*[805428571.4   805428571.4 805428571.4 523528571.4 523528571.4 523528571.4
523528571.4 523528571.4 523528571.4 523528571.4 523528571.4 523528571.4 523528571.4
523528571.4 523528571.4 523528571.4 523528571.4 523528571.4 523528571.4 523528571.4
523528571.4 523528571.4 523528571.4 523528571.4 523528571.4 523528571.4 523528571.4
523528571.4 523528571.4 523528571.4 523528571.4 523528571.4 523528571.4 193302857.1
193302857.1 193302857.1 193302857.1 193302857.1 193302857.1*ones(1,262)];
%EHC=10^3*[636280.9177  636280.9177 636280.9177 413582.5965 413582.5965 413582.5965
413582.5965 413582.5965 413582.5965 413582.5965 413582.5965 413582.5965 413582.5965
413582.5965 413582.5965 413582.5965 413582.5965 413582.5965 413582.5965 413582.5965
413582.5965 413582.5965 413582.5965 413582.5965 413582.5965 413582.5965 413582.5965
413582.5965 413582.5965 413582.5965 413582.5965 413582.5965 413582.5965 152707.4202
152707.4202 152707.4202 152707.4202 152707.4202 152707.4202*ones(1,262)];
%ENOx=10^3*[6517924.974 6517924.974 6517924.974 3258962.487 3258962.487 3258962.487
3258962.487 3258962.487 0   0   0   0   0   0   0   0   0   0   0   0   0   0   0   0   0   0   0   0
0   0   0   0   0   456254.7482 456254.7482 456254.7482 456254.7482 456254.7482
456254.7482*ones(1,262)];
%EPM=10^3*[74496.40255  74496.40255 74496.40255 63321.94217 63321.94217 63321.94217
63321.94217 63321.94217 52147.48179 52147.48179 52147.48179 52147.48179 52147.48179
52147.48179 52147.48179 52147.48179 52147.48179 52147.48179 52147.48179 52147.48179
52147.48179 52147.48179 52147.48179 52147.48179 52147.48179 52147.48179 52147.48179
52147.48179 52147.48179 52147.48179 52147.48179 52147.48179 52147.48179 50657.55374
50657.55374 50657.55374 50657.55374 50657.55374 50657.55374*ones(1,262)];
%ESO2=10^3*[987332.4585 987332.4585 987332.4585 493666.2292 493666.2292 493666.2292
493666.2292 493666.2292 0   0   0   0   0   0   0   0   0   0   0   0   0   0   0   0   0   0   0   0
0   0   0   0   0   0   0   0   0   0   0*ones(1,262)];

% Scenario 3-7
%ECO20=10^3*[805428571.4   805428571.4 805428571.4 523528571.4 523528571.4 523528571.4
523528571.4 523528571.4 523528571.4 523528571.4 523528571.4 523528571.4 523528571.4
193302857.1 193302857.1 193302857.1 193302857.1 193302857.1 193302857.1 193302857.1
193302857.1 193302857.1 193302857.1 144977142.9 144977142.9 144977142.9 144977142.9
144977142.9 144977142.9 144977142.9 144977142.9 144977142.9 144977142.9 96651428.57
96651428.57 96651428.57 96651428.57 96651428.57 96651428.57*ones(1,262)];
%EHC=10^3*[636280.9177  636280.9177 636280.9177 413582.5965 413582.5965 413582.5965
413582.5965 413582.5965 413582.5965 413582.5965 413582.5965 413582.5965 413582.5965
152707.4202 152707.4202 152707.4202 152707.4202 152707.4202 152707.4202 152707.4202
152707.4202 152707.4202 152707.4202 114530.5652 114530.5652 114530.5652 114530.5652
114530.5652 114530.5652 114530.5652 114530.5652 114530.5652 114530.5652 76353.71012
76353.71012 76353.71012 76353.71012 76353.71012 76353.71012*ones(1,262)];
%ENOx=10^3*[6517924.974 6517924.974 6517924.974 3258962.487 3258962.487 3258962.487
3258962.487 3258962.487 3258962.487 3258962.487 3258962.487 3258962.487 3258962.487
456254.7482 456254.7482 456254.7482 456254.7482 456254.7482 456254.7482 456254.7482
456254.7482 456254.7482 456254.7482 912509.4964 912509.4964 912509.4964 912509.4964
```

```
912509.4964 912509.4964 912509.4964 912509.4964 912509.4964 912509.4964 1368764.245
1368764.245 1368764.245 1368764.245 1368764.245 1368764.245*ones(1,262)];
%EPM=10^3*[74496.40255  74496.40255 74496.40255 63321.94217 63321.94217 63321.94217
63321.94217 63321.94217 63321.94217 63321.94217 63321.94217 63321.94217 63321.94217
50657.55374 50657.55374 50657.55374 50657.55374 50657.55374 50657.55374 50657.55374
50657.55374 50657.55374 50657.55374 49167.62568 49167.62568 49167.62568 49167.62568
49167.62568 49167.62568 49167.62568 49167.62568 49167.62568 49167.62568 47677.69763
47677.69763 47677.69763 47677.69763 47677.69763 47677.69763*ones(1,262)];
%ESO2=10^3*[987332.4585 987332.4585 987332.4585 493666.2292 493666.2292 493666.2292
493666.2292 493666.2292 493666.2292 493666.2292 493666.2292 493666.2292 493666.2292 0   0   0
0   0   0   0   0   0   0   0   0   0   0   0   0   0   0   0   0   0   0   0   0   0   0*ones(1,262)];

% Scenario 3-8
%ECO20=10^3*[805428571.4   805428571.4 805428571.4 523528571.4 523528571.4 523528571.4
523528571.4 523528571.4 241628571.4 241628571.4 241628571.4 241628571.4 241628571.4
241628571.4 241628571.4 241628571.4 241628571.4 241628571.4 241628571.4 241628571.4
241628571.4 241628571.4 241628571.4 193302857.1 193302857.1 193302857.1 193302857.1
193302857.1 193302857.1 193302857.1 193302857.1 193302857.1 193302857.1 144977142.9
144977142.9 144977142.9 144977142.9 144977142.9 144977142.9*ones(1,262)];
%EHC=10^3*[636280.9177  636280.9177 636280.9177 413582.5965 413582.5965 413582.5965
413582.5965 413582.5965 190884.2753 190884.2753 190884.2753 190884.2753 190884.2753
190884.2753 190884.2753 190884.2753 190884.2753 190884.2753 190884.2753 190884.2753
190884.2753 190884.2753 190884.2753 152707.4202 152707.4202 152707.4202 152707.4202
152707.4202 152707.4202 152707.4202 152707.4202 152707.4202 152707.4202 114530.5652
114530.5652 114530.5652 114530.5652 114530.5652 114530.5652*ones(1,262)];
%ENOx=10^3*[6517924.974 6517924.974 6517924.974 3258962.487 3258962.487 3258962.487
3258962.487 3258962.487 3258962.487 3258962.487 3258962.487 3258962.487 3258962.487 0   0   0
0   0   0   0   0   0   0   456254.7482 456254.7482 456254.7482 456254.7482 456254.7482
456254.7482 456254.7482 456254.7482 456254.7482 456254.7482 912509.4964 912509.4964
912509.4964 912509.4964 912509.4964 912509.4964*ones(1,262)];
%EPM=10^3*[74496.40255  74496.40255 74496.40255 63321.94217 63321.94217 63321.94217
63321.94217 63321.94217 63321.94217 63321.94217 63321.94217 63321.94217 63321.94217
63321.94217 63321.94217 63321.94217 63321.94217 63321.94217 63321.94217 63321.94217
63321.94217 63321.94217 63321.94217 50657.55374 50657.55374 50657.55374 50657.55374
50657.55374 50657.55374 50657.55374 50657.55374 50657.55374 50657.55374 49167.62568
49167.62568 49167.62568 49167.62568 49167.62568 49167.62568*ones(1,262)];
%ESO2=10^3*[987332.4585 987332.4585 987332.4585 493666.2292 493666.2292 493666.2292
493666.2292 493666.2292 493666.2292 493666.2292 493666.2292 493666.2292 493666.2292 0   0   0
0   0   0   0   0   0   0   0   0   0   0   0   0   0   0   0   0   0   0   0   0   0   0*ones(1,262)];

% Scenario 3-9
%ECO20=10^3*[805428571.4 805428571.4 805428571.4 523528571.4 523528571.4 523528571.4
523528571.4 523528571.4 523528571.4 523528571.4 523528571.4 523528571.4 523528571.4
523528571.4 523528571.4 523528571.4 523528571.4 523528571.4 523528571.4 523528571.4
```

```
523528571.4 523528571.4 523528571.4 523528571.4 523528571.4 523528571.4 523528571.4
523528571.4 523528571.4 523528571.4 523528571.4 523528571.4 523528571.4 193302857.1
193302857.1 193302857.1 193302857.1 193302857.1 193302857.1*ones(1,262)];
%EHC=10^3*[636280.9177  636280.9177 636280.9177 413582.5965 413582.5965 413582.5965
413582.5965 413582.5965 413582.5965 413582.5965 413582.5965 413582.5965 413582.5965
413582.5965 413582.5965 413582.5965 413582.5965 413582.5965 413582.5965 413582.5965
413582.5965 413582.5965 413582.5965 413582.5965 413582.5965 413582.5965 413582.5965
413582.5965 413582.5965 413582.5965 413582.5965 413582.5965 413582.5965 152707.4202
152707.4202 152707.4202 152707.4202 152707.4202 152707.4202*ones(1,262)];
%ENOx=10^3*[6517924.974  6517924.974 6517924.974 3258962.487 3258962.487 3258962.487
3258962.487 3258962.487 3258962.487 3258962.487 3258962.487 3258962.487 3258962.487 0   0   0
0   0   0   0   0   0   0   0   0   0   0   0   0   0   0   0   0   456254.7482 456254.7482
456254.7482 456254.7482 456254.7482 456254.7482*ones(1,262)];
%EPM=10^3*[74496.40255  74496.40255 74496.40255 63321.94217 63321.94217 63321.94217
63321.94217 63321.94217 63321.94217 63321.94217 63321.94217 63321.94217 63321.94217
63321.94217 63321.94217 63321.94217 63321.94217 63321.94217 63321.94217 63321.94217
63321.94217 63321.94217 63321.94217 63321.94217 63321.94217 63321.94217 63321.94217
63321.94217 63321.94217 63321.94217 63321.94217 63321.94217 63321.94217 50657.55374
50657.55374 50657.55374 50657.55374 50657.55374 50657.55374*ones(1,262)];
%ESO2=10^3*[987332.4585  987332.4585 987332.4585 493666.2292 493666.2292 493666.2292
493666.2292 493666.2292 493666.2292 493666.2292 493666.2292 493666.2292 493666.2292 0   0   0
0   0   0   0   0   0   0   0   0   0   0   0   0   0   0   0   0   0   0   0   0   0   0*ones(1,262)];

% Scenario 4-1
%ECO20=10^3*[805428571.4   805428571.4 805428571.4 523528571.4 523528571.4 523528571.4
523528571.4 523528571.4 523528571.4 523528571.4 523528571.4 523528571.4 523528571.4
523528571.4 523528571.4 523528571.4 523528571.4 523528571.4 362442857.1 362442857.1
362442857.1 362442857.1 362442857.1 362442857.1 362442857.1 362442857.1 362442857.1
362442857.1 201357142.9 201357142.9 201357142.9 201357142.9 201357142.9 201357142.9
201357142.9 201357142.9 201357142.9 201357142.9 96651428.57 96651428.57 96651428.57
96651428.57 96651428.57 96651428.57*ones(1,257)];
%EHC=10^3*[636280.9177  636280.9177 636280.9177 413582.5965 413582.5965 413582.5965
413582.5965 413582.5965 413582.5965 413582.5965 413582.5965 413582.5965 413582.5965
413582.5965 413582.5965 413582.5965 413582.5965 413582.5965 286326.413  286326.413
286326.413  286326.413  286326.413  286326.413  286326.413  286326.413  286326.413  286326.413
159070.2294 159070.2294 159070.2294 159070.2294 159070.2294 159070.2294 159070.2294
159070.2294 159070.2294 159070.2294 76353.71012 76353.71012 76353.71012 76353.71012
76353.71012 76353.71012*ones(1,257)];
%ENOx=10^3*[6517924.974 6517924.974 6517924.974 3258962.487 3258962.487 3258962.487
3258962.487 3258962.487 3258962.487 3258962.487 3258962.487 3258962.487 3258962.487
3258962.487 3258962.487 3258962.487 3258962.487 3258962.487 1955377.492 1955377.492
1955377.492 1955377.492 1955377.492 1955377.492 1955377.492 1955377.492 1955377.492
1955377.492 651792.4974 651792.4974 651792.4974 651792.4974 651792.4974 651792.4974
651792.4974 651792.4974 651792.4974 651792.4974 0   0   0   0   0   0*ones(1,257)];
```

```
%EPM=10^3*[74496.40255  74496.40255 74496.40255 63321.94217 63321.94217 63321.94217
63321.94217 63321.94217 63321.94217 63321.94217 63321.94217 63321.94217 63321.94217
63321.94217 63321.94217 63321.94217 63321.94217 63321.94217 57362.22997 57362.22997
57362.22997 57362.22997 57362.22997 57362.22997 57362.22997 57362.22997 57362.22997
57362.22997 51402.51776 51402.51776 51402.51776 51402.51776 51402.51776 51402.51776
51402.51776 51402.51776 51402.51776 51402.51776 47677.69763 47677.69763 47677.69763
47677.69763 47677.69763 47677.69763*ones(1,257)];
%ESO2=10^3*[987332.4585 987332.4585 987332.4585 493666.2292 493666.2292 493666.2292
493666.2292 493666.2292 493666.2292 493666.2292 493666.2292 493666.2292 493666.2292
493666.2292 493666.2292 493666.2292 493666.2292 493666.2292 296199.7375 296199.7375
296199.7375 296199.7375 296199.7375 296199.7375 296199.7375 296199.7375 296199.7375
296199.7375 98733.24585 98733.24585 98733.24585 98733.24585 98733.24585 98733.24585
98733.24585 98733.24585 98733.24585 98733.24585 0   0   0   0   0   0*ones(1,257)];

% Scenario 4-2
%ECO20=10^3*[805428571.4   805428571.4 805428571.4 523528571.4 523528571.4 523528571.4
523528571.4 523528571.4 523528571.4 523528571.4 523528571.4 523528571.4 523528571.4
523528571.4 523528571.4 523528571.4 523528571.4 523528571.4 523528571.4 523528571.4
523528571.4 523528571.4 523528571.4 523528571.4 523528571.4 523528571.4 523528571.4
523528571.4 362442857.1 362442857.1 362442857.1 362442857.1 362442857.1 362442857.1
362442857.1 362442857.1 362442857.1 362442857.1 201357142.9 201357142.9 201357142.9
201357142.9 201357142.9 201357142.9*ones(1,257)];
%EHC=10^3*[636280.9177  636280.9177 636280.9177 413582.5965 413582.5965 413582.5965
413582.5965 413582.5965 413582.5965 413582.5965 413582.5965 413582.5965 413582.5965
413582.5965 413582.5965 413582.5965 413582.5965 413582.5965 413582.5965 413582.5965
413582.5965 413582.5965 413582.5965 413582.5965 413582.5965 413582.5965 413582.5965
413582.5965 286326.413  286326.413  286326.413  286326.413  286326.413  286326.413  286326.413
286326.413  286326.413  286326.413  159070.2294 159070.2294 159070.2294 159070.2294
159070.2294 159070.2294*ones(1,257)];
%ENOx=10^3*[6517924.974 6517924.974 6517924.974 3258962.487 3258962.487 3258962.487
3258962.487 3258962.487 3258962.487 3258962.487 3258962.487 3258962.487 3258962.487
3258962.487 3258962.487 3258962.487 3258962.487 3258962.487 3258962.487 3258962.487
3258962.487 3258962.487 3258962.487 3258962.487 3258962.487 3258962.487 3258962.487
3258962.487 1955377.492 1955377.492 1955377.492 1955377.492 1955377.492 1955377.492
1955377.492 1955377.492 1955377.492 1955377.492 651792.4974 651792.4974 651792.4974
651792.4974 651792.4974 651792.4974*ones(1,257)];
%EPM=10^3*[74496.40255  74496.40255 74496.40255 63321.94217 63321.94217 63321.94217
63321.94217 63321.94217 63321.94217 63321.94217 63321.94217 63321.94217 63321.94217
63321.94217 63321.94217 63321.94217 63321.94217 63321.94217 63321.94217 63321.94217
63321.94217 63321.94217 63321.94217 63321.94217 63321.94217 63321.94217 63321.94217
63321.94217 57362.22997 57362.22997 57362.22997 57362.22997 57362.22997 57362.22997
57362.22997 57362.22997 57362.22997 57362.22997 51402.51776 51402.51776 51402.51776
51402.51776 51402.51776 51402.51776*ones(1,257)];
%ESO2=10^3*[987332.4585 987332.4585 987332.4585 493666.2292 493666.2292 493666.2292
```

```
493666.2292 493666.2292 493666.2292 493666.2292 493666.2292 493666.2292 493666.2292
493666.2292 493666.2292 493666.2292 493666.2292 493666.2292 493666.2292 493666.2292
493666.2292 493666.2292 493666.2292 493666.2292 493666.2292 493666.2292 493666.2292
493666.2292 296199.7375 296199.7375 296199.7375 296199.7375 296199.7375 296199.7375
296199.7375 296199.7375 296199.7375 296199.7375 98733.24585 98733.24585 98733.24585
98733.24585 98733.24585 98733.24585*ones(1,257)];

% Scenario 4-3
%ECO20=10^3*[805428571.4   805428571.4 805428571.4 523528571.4 523528571.4 523528571.4
523528571.4 523528571.4 523528571.4 523528571.4 523528571.4 523528571.4 523528571.4
523528571.4 523528571.4 523528571.4 523528571.4 523528571.4 523528571.4 523528571.4
523528571.4 523528571.4 523528571.4 523528571.4 523528571.4 523528571.4 523528571.4
523528571.4 523528571.4 523528571.4 523528571.4 523528571.4 523528571.4 523528571.4
523528571.4 523528571.4 523528571.4 523528571.4 362442857.1 362442857.1 362442857.1
362442857.1 362442857.1 362442857.1*ones(1,257)];
%EHC=10^3*[636280.9177  636280.9177 636280.9177 413582.5965 413582.5965 413582.5965
413582.5965 413582.5965 413582.5965 413582.5965 413582.5965 413582.5965 413582.5965
413582.5965 413582.5965 413582.5965 413582.5965 413582.5965 413582.5965 413582.5965
413582.5965 413582.5965 413582.5965 413582.5965 413582.5965 413582.5965 413582.5965
413582.5965 413582.5965 413582.5965 413582.5965 413582.5965 413582.5965 413582.5965
413582.5965 413582.5965 413582.5965 413582.5965 286326.413  286326.413  286326.413
286326.413  286326.413  286326.413*ones(1,257)];
%ENOx=10^3*[6517924.974 6517924.974 6517924.974 3258962.487 3258962.487 3258962.487
3258962.487 3258962.487 3258962.487 3258962.487 3258962.487 3258962.487 3258962.487
3258962.487 3258962.487 3258962.487 3258962.487 3258962.487 3258962.487 3258962.487
3258962.487 3258962.487 3258962.487 3258962.487 3258962.487 3258962.487 3258962.487
3258962.487 3258962.487 3258962.487 3258962.487 3258962.487 3258962.487 3258962.487
3258962.487 3258962.487 3258962.487 3258962.487 1955377.492 1955377.492 1955377.492
1955377.492 1955377.492 1955377.492*ones(1,257)];
%EPM=10^3*[74496.40255  74496.40255 74496.40255 63321.94217 63321.94217 63321.94217
63321.94217 63321.94217 63321.94217 63321.94217 63321.94217 63321.94217 63321.94217
63321.94217 63321.94217 63321.94217 63321.94217 63321.94217 63321.94217 63321.94217
63321.94217 63321.94217 63321.94217 63321.94217 63321.94217 63321.94217 63321.94217
63321.94217 63321.94217 63321.94217 63321.94217 63321.94217 63321.94217 63321.94217
63321.94217 63321.94217 63321.94217 63321.94217 57362.22997 57362.22997 57362.22997
57362.22997 57362.22997 57362.22997*ones(1,257)];
%ESO2=10^3*[987332.4585 987332.4585 987332.4585 493666.2292 493666.2292 493666.2292
493666.2292 493666.2292 493666.2292 493666.2292 493666.2292 493666.2292 493666.2292
493666.2292 493666.2292 493666.2292 493666.2292 493666.2292 493666.2292 493666.2292
493666.2292 493666.2292 493666.2292 493666.2292 493666.2292 493666.2292 493666.2292
493666.2292 493666.2292 493666.2292 493666.2292 493666.2292 493666.2292 493666.2292
493666.2292 493666.2292 493666.2292 493666.2292 296199.7375 296199.7375 296199.7375
296199.7375 296199.7375 296199.7375*ones(1,257)];
```

```
% Scenario 4-4
%ECO20=10^3*[805428571.4   805428571.4 805428571.4 523528571.4 523528571.4 523528571.4
523528571.4 523528571.4 241628571.4 241628571.4 241628571.4 241628571.4 241628571.4
241628571.4 241628571.4 241628571.4 241628571.4 241628571.4 193302857.1 193302857.1
193302857.1 193302857.1 193302857.1 193302857.1 193302857.1 193302857.1 193302857.1
193302857.1 144977142.9 144977142.9 144977142.9 144977142.9 144977142.9 144977142.9
144977142.9 144977142.9 144977142.9 144977142.9 96651428.57 96651428.57 96651428.57
96651428.57 96651428.57 96651428.57*ones(1,257)];
%EHC=10^3*[636280.9177  636280.9177 636280.9177 413582.5965 413582.5965 413582.5965
413582.5965 413582.5965 190884.2753 190884.2753 190884.2753 190884.2753 190884.2753
190884.2753 190884.2753 190884.2753 190884.2753 190884.2753 152707.4202 152707.4202
152707.4202 152707.4202 152707.4202 152707.4202 152707.4202 152707.4202 152707.4202
152707.4202 114530.5652 114530.5652 114530.5652 114530.5652 114530.5652 114530.5652
114530.5652 114530.5652 114530.5652 114530.5652 76353.71012 76353.71012 76353.71012
76353.71012 76353.71012 76353.71012*ones(1,257)];
%ENOx=10^3*[6517924.974 6517924.974 6517924.974 3258962.487 3258962.487 3258962.487
3258962.487 3258962.487 0   0   0   0   0   0   0   0   0   0   0   0   0   0   0   0   0   0   0   0
0   0   0   0   0   0   0   0   0   0   0   0   0   0   0   0*ones(1,257)];
%EPM=10^3*[74496.40255  74496.40255 74496.40255 63321.94217 63321.94217 63321.94217
63321.94217 63321.94217 52147.48179 52147.48179 52147.48179 52147.48179 52147.48179
52147.48179 52147.48179 52147.48179 52147.48179 52147.48179 50657.55374 50657.55374
50657.55374 50657.55374 50657.55374 50657.55374 50657.55374 50657.55374 50657.55374
50657.55374 49167.62568 49167.62568 49167.62568 49167.62568 49167.62568 49167.62568
49167.62568 49167.62568 49167.62568 49167.62568 47677.69763 47677.69763 47677.69763
47677.69763 47677.69763 47677.69763*ones(1,257)];
%ESO2=10^3*[987332.4585 987332.4585 987332.4585 493666.2292 493666.2292 493666.2292
493666.2292 493666.2292 0   0   0   0   0   0   0   0   0   0   0   0   0   0   0   0   0   0   0   0
0   0   0   0   0   0   0   0   0   0   0   0   0   0   0   0*ones(1,257)];

% Scenario 4-5
%ECO20=10^3*[805428571.4   805428571.4 805428571.4 523528571.4 523528571.4 523528571.4
523528571.4 523528571.4 523528571.4 523528571.4 523528571.4 523528571.4 523528571.4
523528571.4 523528571.4 523528571.4 523528571.4 523528571.4 523528571.4 523528571.4
523528571.4 523528571.4 523528571.4 523528571.4 523528571.4 523528571.4 523528571.4
523528571.4 193302857.1 193302857.1 193302857.1 193302857.1 193302857.1 193302857.1
193302857.1 193302857.1 193302857.1 193302857.1 144977142.9 144977142.9 144977142.9
144977142.9 144977142.9 144977142.9*ones(1,257)];
%EHC=10^3*[636280.9177  636280.9177 636280.9177 413582.5965 413582.5965 413582.5965
413582.5965 413582.5965 413582.5965 413582.5965 413582.5965 413582.5965 413582.5965
413582.5965 413582.5965 413582.5965 413582.5965 413582.5965 413582.5965 413582.5965
413582.5965 413582.5965 413582.5965 413582.5965 413582.5965 413582.5965 413582.5965
413582.5965 152707.4202 152707.4202 152707.4202 152707.4202 152707.4202 152707.4202
152707.4202 152707.4202 152707.4202 152707.4202 114530.5652 114530.5652 114530.5652
114530.5652 114530.5652 114530.5652*ones(1,257)];
```

```
%ENOx=10^3*[6517924.974 6517924.974 6517924.974 3258962.487 3258962.487 3258962.487
3258962.487 3258962.487 0   0   0   0   0   0   0   0   0   0   0   0   0   0   0   0   0   0   0   0
0   0   0   0   0   0   0   0   0   0   0   0   0   0   0   0*ones(1,257)];
%EPM=10^3*[74496.40255  74496.40255 74496.40255 63321.94217 63321.94217 63321.94217
63321.94217 63321.94217 52147.48179 52147.48179 52147.48179 52147.48179 52147.48179
52147.48179 52147.48179 52147.48179 52147.48179 52147.48179 52147.48179 52147.48179
52147.48179 52147.48179 52147.48179 52147.48179 52147.48179 52147.48179 52147.48179
52147.48179 50657.55374 50657.55374 50657.55374 50657.55374 50657.55374 50657.55374
50657.55374 50657.55374 50657.55374 50657.55374 49167.62568 49167.62568 49167.62568
49167.62568 49167.62568 49167.62568*ones(1,257)];
%ESO2=10^3*[987332.4585 987332.4585 987332.4585 493666.2292 493666.2292 493666.2292
493666.2292 493666.2292 0   0   0   0   0   0   0   0   0   0   0   0   0   0   0   0   0   0   0   0
0   0   0   0   0   0   0   0   0   0   0   0   0   0   0   0*ones(1,257)];

% Scenario 4-6
%ECO20=10^3*[805428571.4   805428571.4 805428571.4 523528571.4 523528571.4 523528571.4
523528571.4 523528571.4 523528571.4 523528571.4 523528571.4 523528571.4 523528571.4
523528571.4 523528571.4 523528571.4 523528571.4 523528571.4 523528571.4 523528571.4
523528571.4 523528571.4 523528571.4 523528571.4 523528571.4 523528571.4 523528571.4
523528571.4 523528571.4 523528571.4 523528571.4 523528571.4 523528571.4 523528571.4
523528571.4 523528571.4 523528571.4 523528571.4 193302857.1 193302857.1 193302857.1
193302857.1 193302857.1 193302857.1*ones(1,257)];
%EHC=10^3*[636280.9177  636280.9177 636280.9177 413582.5965 413582.5965 413582.5965
413582.5965 413582.5965 413582.5965 413582.5965 413582.5965 413582.5965 413582.5965
413582.5965 413582.5965 413582.5965 413582.5965 413582.5965 413582.5965 413582.5965
413582.5965 413582.5965 413582.5965 413582.5965 413582.5965 413582.5965 413582.5965
413582.5965 413582.5965 413582.5965 413582.5965 413582.5965 413582.5965 413582.5965
413582.5965 413582.5965 413582.5965 413582.5965 152707.4202 152707.4202 152707.4202
152707.4202 152707.4202 152707.4202*ones(1,257)];
%ENOx=10^3*[6517924.974 6517924.974 6517924.974 3258962.487 3258962.487 3258962.487
3258962.487 3258962.487 0   0   0   0   0   0   0   0   0   0   0   0   0   0   0   0   0   0   0   0
0   0   0   0   0   0   0   0   0   0   0   0   0   0   0   0*ones(1,257)];
%EPM=10^3*[74496.40255  74496.40255 74496.40255 63321.94217 63321.94217 63321.94217
63321.94217 63321.94217 52147.48179 52147.48179 52147.48179 52147.48179 52147.48179
52147.48179 52147.48179 52147.48179 52147.48179 52147.48179 52147.48179 52147.48179
52147.48179 52147.48179 52147.48179 52147.48179 52147.48179 52147.48179 52147.48179
52147.48179 52147.48179 52147.48179 52147.48179 52147.48179 52147.48179 52147.48179
52147.48179 52147.48179 52147.48179 52147.48179 50657.55374 50657.55374 50657.55374
50657.55374 50657.55374 50657.55374*ones(1,257)];
%ESO2=10^3*[987332.4585 987332.4585 987332.4585 493666.2292 493666.2292 493666.2292
493666.2292 493666.2292 0   0   0   0   0   0   0   0   0   0   0   0   0   0   0   0   0   0   0   0
0   0   0   0   0   0   0   0   0   0   0   0   0   0   0   0*ones(1,257)];

% Scenario 4-7
```

```
%ECO20=10^3*[805428571.4   805428571.4 805428571.4 523528571.4 523528571.4 523528571.4
523528571.4 523528571.4 523528571.4 523528571.4 523528571.4 523528571.4 523528571.4
523528571.4 523528571.4 523528571.4 523528571.4 523528571.4 193302857.1 193302857.1
193302857.1 193302857.1 193302857.1 193302857.1 193302857.1 193302857.1 193302857.1
193302857.1 144977142.9 144977142.9 144977142.9 144977142.9 144977142.9 144977142.9
144977142.9 144977142.9 144977142.9 144977142.9 96651428.57 96651428.57 96651428.57
96651428.57 96651428.57 96651428.57*ones(1,257)];
%EHC=10^3*[636280.9177  636280.9177 636280.9177 413582.5965 413582.5965 413582.5965
413582.5965 413582.5965 413582.5965 413582.5965 413582.5965 413582.5965 413582.5965
413582.5965 413582.5965 413582.5965 413582.5965 413582.5965 152707.4202 152707.4202
152707.4202 152707.4202 152707.4202 152707.4202 152707.4202 152707.4202 152707.4202
152707.4202 114530.5652 114530.5652 114530.5652 114530.5652 114530.5652 114530.5652
114530.5652 114530.5652 114530.5652 114530.5652 76353.71012 76353.71012 76353.71012
76353.71012 76353.71012 76353.71012*ones(1,257)];
%ENOx=10^3*[6517924.974 6517924.974 6517924.974 3258962.487 3258962.487 3258962.487
3258962.487 3258962.487 3258962.487 3258962.487 3258962.487 3258962.487 3258962.487
3258962.487 3258962.487 3258962.487 3258962.487 3258962.487 0   0   0   0   0   0   0   0   0
0   0   0   0   0   0   0   0   0   0   0   0   0   0   0   0   0*ones(1,257)];
%EPM=10^3*[74496.40255  74496.40255 74496.40255 63321.94217 63321.94217 63321.94217
63321.94217 63321.94217 63321.94217 63321.94217 63321.94217 63321.94217 63321.94217
63321.94217 63321.94217 63321.94217 63321.94217 63321.94217 50657.55374 50657.55374
50657.55374 50657.55374 50657.55374 50657.55374 50657.55374 50657.55374 50657.55374
50657.55374 49167.62568 49167.62568 49167.62568 49167.62568 49167.62568 49167.62568
49167.62568 49167.62568 49167.62568 49167.62568 47677.69763 47677.69763 47677.69763
47677.69763 47677.69763 47677.69763*ones(1,257)];
%ESO2=10^3*[987332.4585 987332.4585 987332.4585 493666.2292 493666.2292 493666.2292
493666.2292 493666.2292 493666.2292 493666.2292 493666.2292 493666.2292 493666.2292
493666.2292 493666.2292 493666.2292 493666.2292 493666.2292 0   0   0   0   0   0   0   0   0
0   0   0   0   0   0   0   0   0   0   0   0   0   0   0   0   0*ones(1,257)];

% Scenario 4-8
%ECO20=10^3*[805428571.4   805428571.4 805428571.4 523528571.4 523528571.4 523528571.4
523528571.4 523528571.4 241628571.4 241628571.4 241628571.4 241628571.4 241628571.4
241628571.4 241628571.4 241628571.4 241628571.4 241628571.4 241628571.4 241628571.4
241628571.4 241628571.4 241628571.4 241628571.4 241628571.4 241628571.4 241628571.4
241628571.4 193302857.1 193302857.1 193302857.1 193302857.1 193302857.1 193302857.1
193302857.1 193302857.1 193302857.1 193302857.1 144977142.9 144977142.9 144977142.9
144977142.9 144977142.9 144977142.9*ones(1,257)];
%EHC=10^3*[636280.9177  636280.9177 636280.9177 413582.5965 413582.5965 413582.5965
413582.5965 413582.5965 190884.2753 190884.2753 190884.2753 190884.2753 190884.2753
190884.2753 190884.2753 190884.2753 190884.2753 190884.2753 190884.2753 190884.2753
190884.2753 190884.2753 190884.2753 190884.2753 190884.2753 190884.2753 190884.2753
190884.2753 152707.4202 152707.4202 152707.4202 152707.4202 152707.4202 152707.4202
152707.4202 152707.4202 152707.4202 152707.4202 114530.5652 114530.5652 114530.5652
```

```
114530.5652 114530.5652 114530.5652*ones(1,257)];
%ENOx=10^3*[6517924.974 6517924.974 6517924.974 3258962.487 3258962.487 3258962.487
3258962.487 3258962.487 3258962.487 3258962.487 3258962.487 3258962.487 3258962.487
3258962.487 3258962.487 3258962.487 3258962.487 3258962.487 0   0   0   0   0   0   0   0   0
0   0   0   0   0   0   0   0   0   0   0   0   0   0   0   0   0*ones(1,257)];
%EPM=10^3*[74496.40255  74496.40255 74496.40255 63321.94217 63321.94217 63321.94217
63321.94217 63321.94217 63321.94217 63321.94217 63321.94217 63321.94217 63321.94217
63321.94217 63321.94217 63321.94217 63321.94217 63321.94217 63321.94217 63321.94217
63321.94217 63321.94217 63321.94217 63321.94217 63321.94217 63321.94217 63321.94217
63321.94217 50657.55374 50657.55374 50657.55374 50657.55374 50657.55374 50657.55374
50657.55374 50657.55374 50657.55374 50657.55374 49167.62568 49167.62568 49167.62568
49167.62568 49167.62568 49167.62568*ones(1,257)];
%ESO2=10^3*[987332.4585 987332.4585 987332.4585 493666.2292 493666.2292 493666.2292
493666.2292 493666.2292 493666.2292 493666.2292 493666.2292 493666.2292 493666.2292
493666.2292 493666.2292 493666.2292 493666.2292 493666.2292 0   0   0   0   0   0   0   0   0
0   0   0   0   0   0   0   0   0   0   0   0   0   0   0   0   0*ones(1,257)];

% Scenario 4-9
%ECO20=10^3*[805428571.4 805428571.4 805428571.4 523528571.4 523528571.4 523528571.4
523528571.4 523528571.4 523528571.4 523528571.4 523528571.4 523528571.4 523528571.4
523528571.4 523528571.4 523528571.4 523528571.4 523528571.4 523528571.4 523528571.4
523528571.4 523528571.4 523528571.4 523528571.4 523528571.4 523528571.4 523528571.4
523528571.4 523528571.4 523528571.4 523528571.4 523528571.4 523528571.4 523528571.4
523528571.4 523528571.4 523528571.4 523528571.4 193302857.1 193302857.1 193302857.1
193302857.1 193302857.1 193302857.1*ones(1,257)];
%EHC=10^3*[636280.9177  636280.9177 636280.9177 413582.5965 413582.5965 413582.5965
413582.5965 413582.5965 413582.5965 413582.5965 413582.5965 413582.5965 413582.5965
413582.5965 413582.5965 413582.5965 413582.5965 413582.5965 413582.5965 413582.5965
413582.5965 413582.5965 413582.5965 413582.5965 413582.5965 413582.5965 413582.5965
413582.5965 413582.5965 413582.5965 413582.5965 413582.5965 413582.5965 413582.5965
413582.5965 413582.5965 413582.5965 413582.5965 152707.4202 152707.4202 152707.4202
152707.4202 152707.4202 152707.4202*ones(1,257)];
%ENOx=10^3*[6517924.974  6517924.974 6517924.974 3258962.487 3258962.487 3258962.487
3258962.487 3258962.487 3258962.487 3258962.487 3258962.487 3258962.487 3258962.487
3258962.487 3258962.487 3258962.487 3258962.487 3258962.487 0   0   0   0   0   0   0   0   0
0   0   0   0   0   0   0   0   0   0   0   0   0   0   0   0   0*ones(1,257)];
%EPM=10^3*[74496.40255  74496.40255 74496.40255 63321.94217 63321.94217 63321.94217
63321.94217 63321.94217 63321.94217 63321.94217 63321.94217 63321.94217 63321.94217
63321.94217 63321.94217 63321.94217 63321.94217 63321.94217 63321.94217 63321.94217
63321.94217 63321.94217 63321.94217 63321.94217 63321.94217 63321.94217 63321.94217
63321.94217 63321.94217 63321.94217 63321.94217 63321.94217 63321.94217 63321.94217
63321.94217 63321.94217 63321.94217 63321.94217 50657.55374 50657.55374 50657.55374
50657.55374 50657.55374 50657.55374*ones(1,257)];
%ESO2=10^3*[987332.4585  987332.4585 987332.4585 493666.2292 493666.2292 493666.2292
```

```
493666.2292 493666.2292 493666.2292 493666.2292 493666.2292 493666.2292 493666.2292
493666.2292 493666.2292 493666.2292 493666.2292 493666.2292 0   0   0   0   0   0   0   0   0
0   0   0   0   0   0   0   0   0   0   0   0   0   0   0   0   0*ones(1,257)];

alpha=0.4;%The proportion of CH4 in HC
ECH4=alpha*EHC;
ZHUANHUA=0.2; %The proportion conversion ratio of CH4 to CO2
ECO2CH4=0.61*ZHUANHUA*ECH4*(1-exp(-1/9.3)); % The period of CH4 is 9.3 years
ECO2=ECO20+ECO2CH4;
a0=0.25;
a1=0.25;
a2=0.25;
a3=1-a0-a1-a2;
beta=unifrnd(10000000,805428571,300,10^6);%one hundred thousand Monte Carlo simulations
CCO2=0; % The original concentration of CO2 is 0

%The process to calculate the concentration of CO2
R0=10000000*ones(1,300);
R1=10000000*ones(1,300);
R2=10000000*ones(1,300);
R3=10000000*ones(1,300);
for t=2:300
   for i=1:10^6
      R0(t)=a0*ECO2(t)-R0(t-1)/beta(t,i)+R0(t-1);
      R1(t)=a1*ECO2(t)-R1(t-1)/beta(t,i)+R1(t-1);
      R2(t)=a2*ECO2(t)-R2(t-1)/beta(t,i)+R2(t-1);
      R3(t)=a3*ECO2(t)-R3(t-1)/beta(t,i)+R3(t-1);
      CCO2(t)=278+44.095/(28.966*5.1352*10^(18))*(R0(t)+R1(t)+R2(t)+R3(t));
      Cacc(t)=sum(ECO2(1:t))-(CCO2(t)-278);
      if abs(beta(t,i)*(1-exp(-100/beta(t,i)))-35-0.019*Cacc(t)-4.165*1.2)>eps
         i=i+1;
      end
   end
end
  % CCO2 is the concentration of CO2

%The process to calculate the concentration of CH4
ECH4=alpha*EHC;
ZHUANHUA=0.2;
CCCH4=zeros(310,300);
CCH4=zeros(1,300);
for t=2:290
   ECH4(t)=(1-ZHUANHUA)*ECH4(t);
   for i=t-1:t+9
```

```
        CCCH4(i,t)=CCCH4(i,t-1)+1/2*(ECH4(t-
1)/(5.1352*10^(18))*28.966/44.013+ECH4(t)/(5.1352*10^(18))*28.966/16.04)-CCCH4(i,t-1)*(1-
exp(-1/9.3));
    end
     CCH4(t)=sum(CCCH4(t,:));
end
  % CCH4 is the concentration of CH4

 %The process to calculate the concentration of N2O

gema=0.29;% The proportion of N2O in NOx
CCN2O=zeros(421,300); % The period of CH4 is 121 years, so the total is 300+121=421
CN2O=zeros(1,300); % The CN2O in 2022 is 0
for t=2:300
    EN2O(t)=gema*ENOx(t);
    for i=t-1:t+120
        CCN2O(i,t)=CCN2O(i,t-1)+1/2*(EN2O(t-
1)/(5.1352*10^(18))*28.966/44.013+EN2O(t)/(5.1352*10^(18))*28.966/44.013)-CCN2O(i,t-1)*(1-
exp(-1/121));
    end
        CN2O(t)=0.1*sum(CCN2O(t,:));
end
 % CN2O is the concentration of N2O

%The process to calculate the ERF
 FCO2=zeros(1,300);% The ERF of CO2
 FN2O=zeros(1,300); % The ERF of N2O
 FCH4=zeros(1,300);% The ERF of CH4
 Fair=(0.08*EPM-0.34*ESO2-0.044*ENOx)/(5.1352*10^(18)); % The ERF of Aerosols
 Fcon=0.0152*ENOx/(5.1352*10^(18)); % The ERF of Contrails
 for t=1:300
     FCO2(t)=[(-2.4*10^(-7))*(CCO2(t)-278)^2+7.2*10^(-4)*abs(CCO2(t)-278)-1.05*10^(-
4)*CN2O(t)+5.36]*log(CCO2(t)/278);
     FN2O(t)=[(-4*10^(-6))*(CCO2(t)+278)+2.1*10^(-6)*CN2O(t)-2.45*10^(-
6)*CCH4(t)+0.117]*sqrt(CN2O(t));
     FCH4(t)=[(-6.5*10^(-7))*CCH4(t)-(4.1*10^(-6))*CN2O(t)+0.043]*sqrt(CCH4(t));
 end

 % The process to calculate the temperature change
 T1=zeros(1,300);
 T2=zeros(1,300);
 for t=2:300
     T1(t)=T1(t-
1)*exp(1/239)+0.000387286469545131*(FCO2(t)+FN2O(t)+FCH4(t)+Fair(t)+Fcon(t))*(1-
```

```
exp(1/239));
    T2(t)=T2(t-1)*exp(1/4.1)-
0.0000383603018325489*(FCO2(t)+FN2O(t)+FCH4(t)+Fair(t)+Fcon(t))*(1-exp(1/4.1));
    T(t)=T1(t)+T2(t);
 End
```

References:

1. Bergero C, Gosnell G, Gielen D, et al. Pathways to net-zero emissions from aviation. Nature Sustainability, 1-11 (2023).

2. Marszałek N, Lis T. The future of sustainable aviation fuels. Combustion Engines, 61(4), 29-40 (2022).

3. Airbus. Sustainable aviation fuel--A proven alternative fuel for immediate CO_2 reduction. https://www.airbus.com/en/sustainability/respecting-the-planet/decarbonisation/sustainable-aviation-fuel (2023).

4. Choi, Y and Lee, J. Estimation of Liquid Hydrogen Fuels in Aviation. Aerospace. 9, 564 (2022).

5. McKinsey & Company. Hydrogen-powered aviation: A fact-based study of hydrogen technology, economics, and climate impact by 2050 (2020).
